# Mismatch discrimination and sequence bias during end-joining by DNA ligases

**DOI:** 10.1093/nar/gkac241

**Published:** 2022-04-19

**Authors:** Katharina Bilotti, Vladimir Potapov, John M Pryor, Alexander T Duckworth, James L Keck, Gregory J S Lohman

**Affiliations:** Research Department, New England Biolabs, Ipswich, MA 01938, USA; Research Department, New England Biolabs, Ipswich, MA 01938, USA; Research Department, New England Biolabs, Ipswich, MA 01938, USA; Department of Biomolecular Chemistry, University of Wisconsin School of Medicine and Public Health, Madison, WI 53706, USA; Department of Biomolecular Chemistry, University of Wisconsin School of Medicine and Public Health, Madison, WI 53706, USA; Research Department, New England Biolabs, Ipswich, MA 01938, USA

## Abstract

DNA ligases, critical enzymes for *in vivo* genome maintenance and modern molecular biology, catalyze the joining of adjacent 3′-OH and 5′-phosphorylated ends in DNA. To determine whether DNA annealing equilibria or properties intrinsic to the DNA ligase enzyme impact end-joining ligation outcomes, we used a highly multiplexed, sequencing-based assay to profile mismatch discrimination and sequence bias for several ligases capable of efficient end-joining. Our data reveal a spectrum of fidelity and bias, influenced by both the strength of overhang annealing as well as sequence preferences and mismatch tolerances that vary both in degree and kind between ligases. For example, while T7 DNA ligase shows a strong preference for ligating high GC sequences, other ligases show little GC-dependent bias, with human DNA Ligase 3 showing almost none. Similarly, mismatch tolerance varies widely among ligases, and while all ligases tested were most permissive of G:T mismatches, some ligases also tolerated bulkier purine:purine mismatches. These comprehensive fidelity and bias profiles provide insight into the biology of end-joining reactions and highlight the importance of ligase choice in application design.

## INTRODUCTION

DNA ligases are essential enzymes in genome replication and repair processes, including Okazaki fragment maturation and the repair of nicks left after base and nucleotide excision repair. DNA ligases catalyze the formation of a phosphodiester bond between the 3′-hydroxyl of one DNA strand and the 5′-phosphorylated termini of another DNA strand (1,2). Despite a lack of primary sequence homology between the enzymes, the overall structure of DNA ligases is fairly well conserved (3). All known DNA ligases contain a nucleotidyl transferase domain (NTase), which contains the catalytic lysine residue, and an oligonucleotide binding domain (OBD) that contains a DNA binding surface. X-ray crystal structures for several DNA ligases show a third domain which allows the ligase to completely encircle the DNA substrate, though the presence and character of this domain varies between ligases (4). For example, the third domain is completely lacking from T7 DNA ligase, and this enzyme does not completely encircle the DNA substrate (5). *Paramecium bursaria* chlorella virus 1 (PBCV-1) DNA ligase has a third ‘latch’ domain that protrudes from the OB domain and makes contacts with the NTase domain to encircle its substrate (6). In other crystalized DNA ligases such as T4 DNA ligase, a third, N-terminal DNA binding domain (DBD) has been observed to play this role (7). In some cases, blunt and cohesive-end sealing activity has been shown to require the presence of specific DNA binding domains in the ligase. For example, a unique N-terminal poly ADP-ribose polymerase-like zinc finger domain has been implicated in the ability of human DNA Ligase 3 (hLig3) to carry out non-homologous end-joining without additional protein factors (8–11).

While most DNA ligases have strong activity on substrates containing a single strand break in one strand of a duplex (nick ligation), only a few DNA ligases efficiently join two DNA fragments with short complementary overhangs or blunt ends in the absence of accessory proteins (11–13). This end-joining activity is of critical importance in biotechnology applications, including cloning, molecular diagnostics, DNA assembly, and next-generation sequencing library preparation, which rely on the faithful and efficient end-joining activity of DNA ligases. The most commonly used ligase in these protocols is T4 DNA ligase, but a number of other DNA ligases have also been used. For example, T3 DNA ligase is sometimes favored due to higher salt tolerance than T4 DNA ligase (14), while T7 DNA ligase is capable of high-specificity for cohesive end-joining over blunt end DNA fragments (15). Recently, (PBCV-1) DNA ligase, a DNA ligase that can efficiently join structures with short cohesive ends (16), has found a niche use in diagnostic applications due to a surprising efficiency in ligating DNA-RNA hybrid substrates (17).

Previous investigations into the sequence specificity and discrimination against substrates containing mismatched base-pairs near the ligation junction (fidelity) of DNA ligases have primarily focused on the context of nick ligation (4,18–23). These studies typically used defined sequences examined individually or in small pools of mixed sequences, and assessed the fidelity profile of the enzyme via differential rates of reaction. Although the exact mechanism of ligase fidelity is not known, studies suggest that mismatched bases distort the DNA helix and interfere with minor groove interactions between the DNA and the ligase, disrupting catalytic efficiency via a combination of weakened binding and a disordered active site (24,25). This results in inefficient ligation and frequent abortive adenylation (18). In these studies of nick sealing, ligases generally discriminate more strongly against bulky purine:purine mismatches, which significantly distort the helix, and are more permissive to smaller pyrimidine:pyrimidine and pyrimidine:purine mismatches. Further, in the context of nick ligation, ligases discriminate more stringently against mismatches at the 3′-hydroxyl side of the ligation junction than the 5′-phosphate side (18,26,27). Although general trends for nick ligation fidelity have been determined, many *in vitro* applications rely on ligation of DNA fragments with short complementary ends, and comprehensive information about DNA ligase fidelity in end-joining reactions is currently limited.

There has also been limited inquiry into the sequence preferences of DNA ligases. For example, in the context of nick ligation, T4 DNA ligase displays a preference for nicks where the 5′-phosphorylated base is a pyrimidine compared to a purine, reflected as a two-fold increase in ligation rate (28). We also previously investigated the end-joining activity of DNA ligases on substrates with varying end structure and length (29). Each ligase tested had different end structure preferences, with some ligases limited to ligation of four-base cohesive ends and other ligases able to catalyze blunt end-joining or single base overhang ligation. Further, the preferred substrate between blunt, 3′ single base overhangs, and 5′ single base overhangs varied by ligase. Additionally, while macromolecular crowding reagents, such as polyethylene glycol (PEG), are commonly used as ligation enhancers to boost yield of ligation product, the addition of PEG did not change the relative substrate preferences of a given ligase. As these differences in end structure preference could not be explained by differences in substrate annealing strength alone, we hypothesized that differences in the active site structure of individual DNA ligases have a significant impact on substrate preference in end-joining. However, as this analysis was limited to a single substrate, the sequence biases of DNA ligases in end-joining remain unknown.

We previously reported a highly multiplexed, single molecule sequencing method that permits comprehensive profiling of sequence bias and mismatch tolerance in cohesive end ligation (30). In this assay, we prepared sequencing libraries by mixing DNA ligase with a substrate containing degenerate overhangs, allowing for every possible end-joining sequence context to be observed in a single reaction (Supplementary Figure S1). Following the ligation reaction, the libraries were sequenced using PacBio Single-Molecule Real-Time (SMRT) sequencing to determine the relative ligation frequency of each overhang and the percentage of correct (Watson–Crick) vs incorrect (mismatch) ligations. We have previously used this method to analyze three- and four-base overhang ligation by T4 DNA ligase. Broadly, we observed low overhang sequence bias, with T4 DNA ligase forming ligation products with most overhang sequences with approximately equal frequency (though with the notable exception of the poorly ligated TNA and TNNA overhangs). Some sequences were faithfully ligated to their Watson–Crick partner, while others could be ligated to a range of mismatched overhangs, dominated by G:T mismatches. Unlike a simple two-pairing ligation reaction, this assay system represents a competition between all possible overhang sequence pairings. We previously showed that the results predict Golden Gate Assembly outcomes extremely accurately, demonstrating that this model system provides accurate predictions of reduced complexity ligation systems (31).

In the current study, we have expanded our previously described method to examine and compare the four-base overhang ligation profiles of T3 DNA ligase, T4 DNA ligase, PBCV-1 DNA ligase, hLig3 and T7 DNA ligase. These ligases have robust end-joining activity in the absence of macromolecular enhancers and are used in a diverse set of biotechnology applications. We observed trends that highlight the differences and similarities between DNA ligases and allow dissection of the effects of substrate annealing versus intrinsic bias and fidelity differences between ligases.

## MATERIALS AND METHODS

All enzymes (excepting hLig3) and buffers were obtained from New England Biolabs (NEB, Ipswich, MA). T4 DNA ligase reaction buffer (1×) is: 50 mM Tris–HCl (pH 7.5), 10 mM MgCl_2_, 1 mM ATP, 10 mM DTT. NEBNext^®^ Quick Ligation reaction buffer (1×) is: 66 mM Tris (pH 7.6), 10 mM MgCl_2_, 1 mM DTT, 1 mM ATP, 6% Polyethylene glycol (PEG 6000). NEBuffer 2 (1×) is: 10 mM Tris–HCl (pH 7.9), 50 mM NaCl, 10 mM MgCl_2_, 1 mM DTT. CutSmart Buffer (1×) is: 20 mM Tris-acetate (pH 7.9), 50 mM potassium acetate, 10 mM magnesium acetate, 100 μg/ml BSA. Thermopol buffer is: 20 mM Tris–HCl (pH 8.8), 10 mM (NH_4_)_2_SO_4_, 10 mM KCl, 2 mM MgSO_4_, 0.1% Triton-X-100. Standard *Taq* polymerase buffer is: 10 mM Tris–HCl (pH 8.3), 50 mM KCl, 1.5 mM MgCl_2_. All column cleanup of oligonucleotides and ligated libraries was performed using Monarch PCR & DNA Cleanup Kit columns (NEB), following the Oligonucleotide Cleanup Protocol. Oligonucleotide purity and sizing was performed using an Agilent Bioanalyzer 2100, using a DNA 1000 assay, following the standard protocols.

The hLig3 beta gene was synthesized by Biomatik (Cambridge, Ontario) and subcloned into a pET28 plasmid in frame with an N-terminal His_6_-tag. The construct was expressed in T7 Express *lysY/I^q^ Escherichia coli* cells (NEB) using 0.5 mM IPTG for 2 h at 30°C for induction. The cell pellet was resuspended in breakage buffer containing 20 mM Tris–HCl (pH 7.5), 300 mM NaCl, 10% glycerol and 1 mM PMSF and stored at −20°C. Cells were thawed and lysed by two passes through a microfluidizer, then clarified by centrifugation at 12,000 RPM for 60 m at 4°C. All subsequent steps were also carried out at 4°C. The clarified lysate was then loaded onto a nickel column equilibrated with 20 mM Tris–HCl (pH 7.5), 300 mM NaCl, 10% glycerol and eluted with a linear gradient of 0–500 mM imidazole. Fractions containing hLig3 were determined by SDS PAGE analysis, pooled, and the salt content of the buffer was lowered to 150 mM NaCl using 20mM Tris–HCl (pH 7.5), 10% glycerol. The enzyme pool was loaded onto a Heparin column and eluted with a linear gradient from 0–1M NaCl. Fractions containing hLig3 were again determined by SDS PAGE analysis and pooled. Following this, the ligase pool was again brought to a salt concentration of 150 mM NaCl before passage over a Q Sepharose anion exchange column equilibrated with 20mM Tris–HCl (pH 7.5), 10% glycerol. hLig3 eluted in the flow through and was passed over a second nickel column for further refinement before a final size exclusion step using a HiLoad 26/60 Superdex column (GE Healthcare) equilibrated with 20 mM Tris (pH 7.5), 300 mM NaCl, 10% glycerol. Fractions containing purified hLig3 (determined by SDS PAGE) were concentrated using Amicon Ultra-15 Centrifugal Filters (MWCO 50 kDa, Millipore Sigma) and stored at −20°C in 20mM Tris–HCl (pH 7.5), 300 mM NaCl, 50% glycerol, 1 mM DTT. The concentration of hLig3 was determined by measuring the UV absorbance at 280 nm.

### Preparation and Pacific Biosciences SMRT sequencing of ligation fidelity libraries

The substrate for the four-base overhang ligation fidelity assay was produced using the previously published protocol (31). Briefly, the initial PAGE-purified substrate precursor oligonucleotide contained a 5′-terminal region, a randomized four-base region, a BsaI-HFv2 binding site, a constant region, an internal 6-base randomized region as a control for synthesis bias, and a region corresponding to the SMRT-bell sequencing adapter for Pacific Biosciences SMRT sequencing. The precursor oligonucleotide was extended as described previously and purified using the Monarch PCR & DNA Cleanup Kit.

The extended DNA was cut using BsaI-HFv2 to generate a four-base overhang as described previously (31). Final concentration and extent of cutting was determined by Agilent Bioanalyzer (DNA 1000) and confirmed to be >95% cut. Remaining uncut starting material (∼5%) was not 5′-phosphorylated and thus should not interfere with subsequent cohesive end-joining reactions. The final substrate sequence can be found in the Supplementary Data (Supplementary Table S1).

For each ligation reaction, substrate (100 nM) was combined with the DNA ligase (either T4 DNA ligase, T3 DNA ligase, T7 DNA ligase, PBCV-1 DNA ligase, or hLig3 at 1.75 μM final concentration) in 1× T4 DNA ligase buffer (or NEBNext^®^ Quick Ligation reaction buffer for reactions noted as containing PEG) in a 50 μl total reaction volume and incubated for 1 h at 25°C. Reactions were quenched with 2.5 μl of ligase reaction quench (500 mM EDTA + 2.5% v/v Proteinase K) and the sample was heated to 37°C for 30 m to allow for ligase cleavage by Proteinase K DNA. The reaction was then purified using the Monarch PCR & DNA Cleanup Kit (NEB) and following the Oligonucleotide Cleanup protocol. Each ligation was performed in a minimum of duplicates, and the ligation yield was determined by Agilent Bioanalyzer (DNA 1000) with error reported as one standard deviation. The ligation yield was calculated using the following equation: Yield = [product]/[substrate + product] × 100.

The ligated library was treated with Exonuclease III (50 U) and Exonuclease VII (5 U) in a 50 μl volume in 1× Standard Taq Polymerase buffer incubated for 1 h at 37°C. The library was purified using a Monarch PCR & DNA Cleanup Kit, oligonucleotide cleanup protocol, including a second wash step, and then quantified by Agilent Bioanalyzer (DNA 1000). Typical concentrations of final library were between 0.5 and 2 ng/μl. Two replicate experiments were conducted for each ligase. Sequencing and analysis of sequencing data were performed as previously described (30). In short, consensus sequences for the top and bottom strand of the ligation product were generated, and actual overhang sequences in each strand were extracted. Frequencies of all observed overhang pairs in ligation products were tabulated and used to derive results. Data from replicates were combined before subsequent analysis. Full results from each experiment can be found in the Supplementary Data (Supplementary Table S2, Supplementary Figures S2A–H and File S1).

### Oligonucleotide ligation assay

Oligo substrate pairs with defined 5′-four base overhangs of varying GC content were ordered from IDT. Oligo sequences can be found in Supplementary Table S3. To create each substrate, the FAM-labeled oligo was annealed to an unlabeled complementary strand (present at a 1.1 molar excess) in 10 mM Tris–HCl, 50 mM NaCl, 0.1 nM EDTA. Substrate annealing resulted in pairs of double stranded oligos with complementary 5′-four base overhangs. Standard ligation assay mixtures were composed of 1× T4 DNA Ligase reaction buffer, 1 nM DNA ligase, and 100 nM FAM-labeled complementary DNA substrates in a reaction volume of 100 μl. Components were gently mixed by pipetting and incubated at 25°C for 5 m prior to initiation by the addition of the DNA substrate. Reactions were quenched by a 1:1 (vol/vol) addition of ligation stop solution (50 mM EDTA, 0.1% Triton X-100) at times as indicated in each figure legend. For reactions with hLig3, Proteinase K was added after quenching (2.5% vol/vol) and the reaction was incubated for 20 m at 37°C. The ligated products were analyzed by capillary gel electrophoresis as described previously (27). Initial velocity rates were obtained by fitting the linear portion of the data (up to a maximum of 25% product conversion) to a linear regression using GraphPad Prism (Supplementary Table S4). Reported values are the average of four replicates, with the error values representing the reported error in the slope of the fit (GraphPad Prism 9.3.0).

## RESULTS

### DNA ligases exhibited varying efficiency and sequence bias

To determine the fidelity and bias profiles of DNA ligases in end-joining, we prepared sequencing libraries by mixing each DNA ligase (T4 DNA Ligase, T3 DNA Ligase, T7 DNA Ligase, PBCV-1 DNA Ligase and hLig3) with a hairpin substrate containing degenerate 5′-four-base overhang ends, allowing for every possible sequence context to be observed in a single reaction (30). The ligase was present in a large excess compared to substrate, similar to the concentration of reactants in standard molecular biology protocols to permit rapid ligation of short cohesive ends. Following the ligation reaction, the libraries were sequenced using PacBio SMRT sequencing and a summary of multiplex ligation data for each ligase, including the total number of ligation events, percentage of correct (Watson–Crick) versus incorrect (mismatch) ligations, and yield of ligation product is in Supplementary Table S2.

Notably, this substrate presents a complex equilibrium system wherein ligation requires finding compatible ends. Therefore, while we would expect a very rapid conversion to ligated product if there were only two Watson–Crick binding partners in the reaction, the assay here presents competing annealing partners in a complex equilibrium, and ligation yields may be limited by the presence of annealed pairings that ligate with poor efficiency. Ultimately, however, the experimental setup provides depth of information not available by separately examining individual overhangs, and permits a much more rapid appraisal of fidelity and bias than possible through testing each pairing in parallel. Library ligation yields at 1 h varied significantly among ligases tested (Supplementary Table S2). T4 DNA ligase, T3 DNA ligase, and hLig3 all yielded greater than 55% ligation product, consistent with our previous observations that these are generally among the most efficient end-joining ligases and the frequent use of T4 DNA ligase and T3 DNA ligase in end-joining applications (29). PBCV-1 ligase had a slightly lower yield (50%), reflecting its less robust end-joining activity. T7 DNA ligase had by far the lowest yield, only reaching 20% ligation product. We also tested the thermostable *Taq* DNA ligase but did not obtain detectable yields of ligated library in this system.

**Figure 1.**
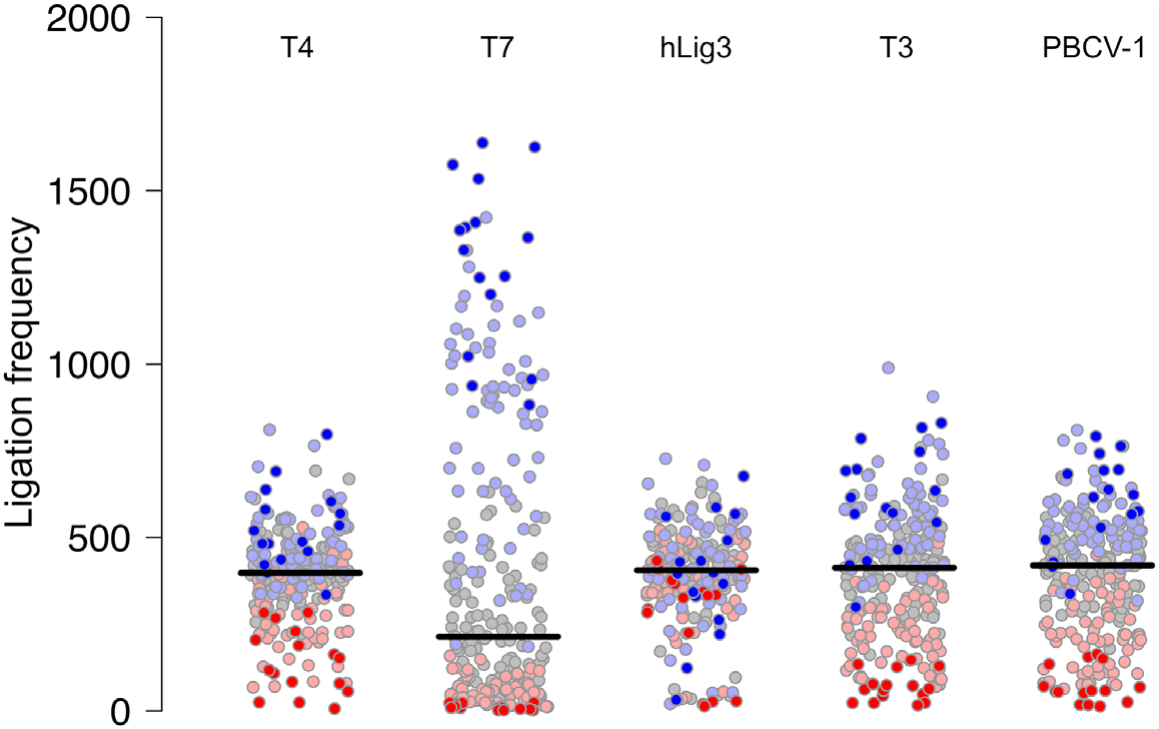
Ligation bias for T4 DNA ligase, T7 DNA ligase, hLig3, T3 DNA ligase and PBCV-1 DNA ligase. The normalized ligation frequency of each overhang was generated by SMRT sequencing of ligation reactions with 100 nM of the multiplexed four-base overhang substrate and 1.75 μM T4 DNA ligase, T7 DNA ligase, hLig3, T3 DNA ligase, or PBCV-1 DNA ligase incubated 1h at 25°C in standard ligation buffer (Supplementary Figures S2A-S2E and File S1 for raw data).T7 DNA ligase exhibits a significant ligation bias with few overhangs ligated very efficiently, and the majority of overhangs ligated with much lower efficiency. T4 DNA ligase and hLig3 exhibit more uniform ligation bias compared to T7 DNA ligase. GC-rich overhangs tend to ligate more efficiently compared to AT-rich overhangs. Each overhang is colored according to its GC content (0% – dark red, 25% – light red, 50% – gray, 75% – light blue, 100% – dark blue.

Analysis of multiplex ligation data illuminated ligation sequence bias in the preferred overhang sequences. In our assay, the number of reads for each overhang is a proxy for its ligation efficiency; the sequence bias for each ligase is inferred from the relative frequency of each overhang appearing across all ligation products. Interestingly, we observed both varying overall degrees of bias, as well as intrinsically different preferred sequences between ligases (Figure 1). T7 DNA ligase showed the highest degree of sequence bias. All other ligases examined had a much tighter distribution of ligation frequencies, but with differences in how tightly the data points are clustered around the average. Both T4 DNA ligase and hLig3 showed the least amount of bias with the range of values >2-fold smaller compared to T7 DNA ligase. PBCV-1 and T3 had a similar average ligation frequency but a slightly larger range of observed ligation frequencies.

**Figure 2.**
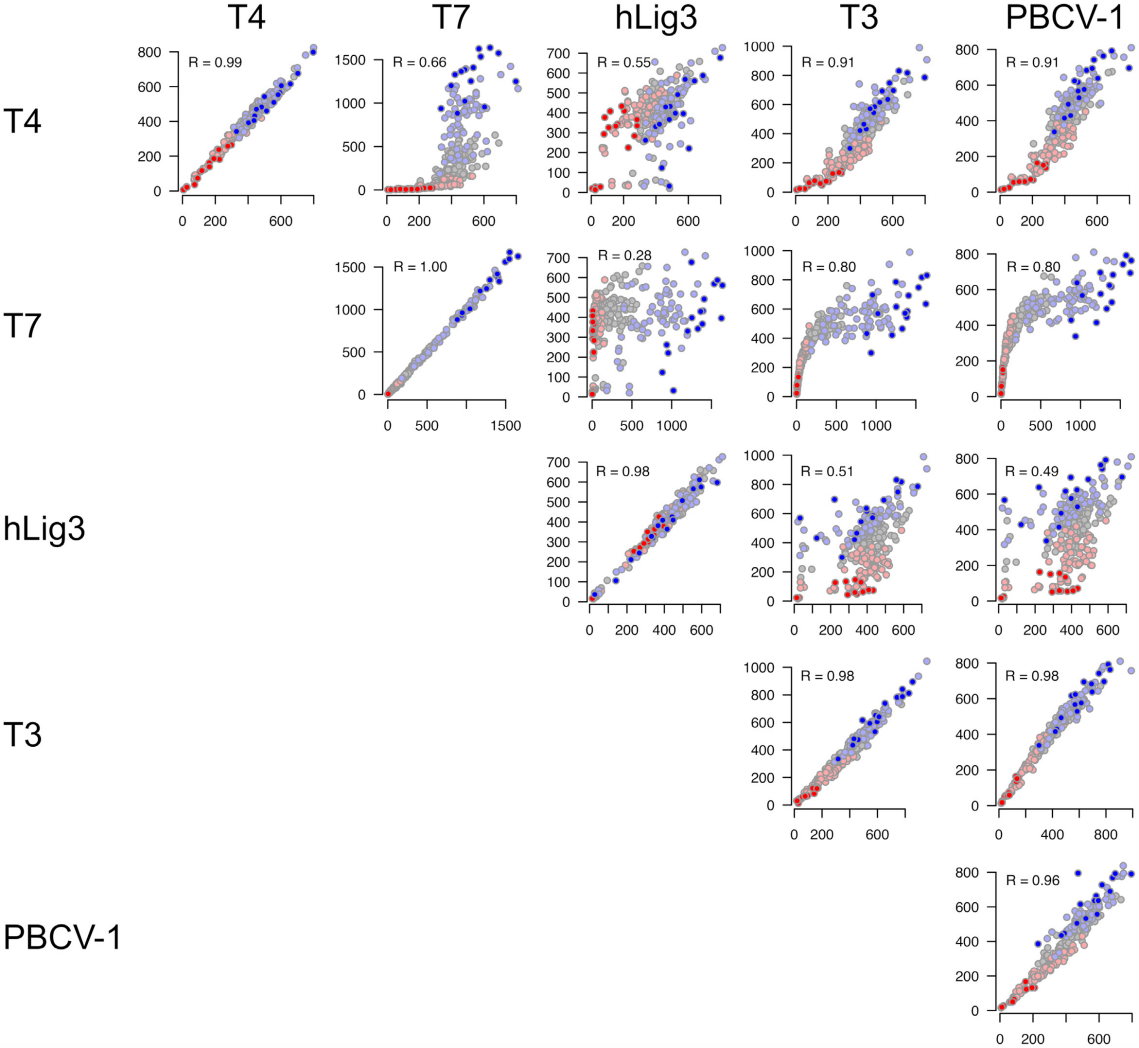
Pairwise comparison of sequence preferences for different ligases. The plots on the diagonal show agreement between ligation bias for two independent replicates for the same ligase. Off-diagonal plots highlight differences in ligation bias for different ligases. Each dot in the plot corresponds to an overhang, and X- and Y- values correspond to ligation frequency of a given overhang in two samples. The individual overhangs are colored according to their GC content (0% – dark red, 25% – light red, 50% – gray, 75% – light blue, 100% – dark blue). Pearson correlation coefficient is reported for each comparison.

Further, when the ligation frequencies of individual overhangs were analyzed, the specific sequences that were preferred or disfavored varied between the enzymes. For most ligases, a weak general trend disposing higher GC content overhangs to more efficient ligation was observed (Figure 1). The bias in favor of high GC pairings was seen for both Watson–Crick ligations and pairings containing at least one mismatch, indicating a preference for more strongly annealed sequences in both cases. T7 DNA ligase was most vulnerable to this bias, with low GC overhangs (0% or 25% GC content) rarely ligated and high GC content (>50%) accounting for 96% of ligated products. These data indicate that for T7 DNA ligase, end-joining ligation efficiency is dominated by the GC content of the overhang. T4 DNA ligase, T3 DNA ligase, and PBCV-1 showed a less pronounced, but still observable dependence on GC content. Conversely, hLig3 ligation appears to be independent of GC content. Clearly, however, GC content is not the only factor contributing to differences in bias. While experimental replicates of each individual enzyme are consistent in the preferred overhang sequences, comparison of sequence preferences between different DNA ligases reveals additional complex differences which cannot be easily described by GC content or other simple trends (Figure 2).

To further explore the dependence of sequence bias on GC content, the initial velocity of ligation was measured using fluorescently labeled dsDNA substrates with defined 5′ four base overhangs of varying GC content (ATAA/TATT, CTAT/ATAG, GTGA/TCAC, AGCG/CGCT, CGGC/GCCG). As a range of ligation frequency was observed among overhangs with each possible GC content, representative overhangs that did not display obvious outlier behavior for any ligase were chosen (Supplementary Table S3). Unlike the multiplex sequencing assay, which uses an excess of DNA ligase over DNA substrate, initial velocity experiments with defined oligos used an excess of substrate (100 nM) over DNA ligase (1 nM). The results of the timecourse experiments and a summary of the initial velocity for each substrate with T4 DNA ligase, T7 DNA ligase, and hLig3 can be found in Supplementary Figure S3 and Supplementary Table S4, respectively. For all ligases, rates increased with increasing overhang GC content, with the exception that hLig3 and T4 Ligase showed a maximum rate for the 75% GC substrate and a decline for 100% GC. For hLig3, which showed minimal influence of GC content on sequence bias in the multiplex assay, the initial velocity on the slowest overhang (0% GC) was only 10-fold slower than with the fastest overhang (75% GC). For T4 DNA ligase, which showed a moderate influence of GC content on sequence bias in the multiplex assay, the initial velocity with the slowest overhang (0% GC) was 100-fold slower than with the fastest overhang (75% GC). Finally, T7 DNA ligase showed the most extreme influence of overhang GC content on sequence bias in the multiplex assay. In the defined oligo assay, the initial velocity of T7 DNA ligase with the slowest overhang (0% GC) was 1000-fold slower than the with the fastest overhang (100% GC). Broadly, the qualitative biases seen in the multiplex assay correlate with the observed initial velocities for each GC content bin. The ligase with the most extreme bias showed the largest difference in between the fastest and slowest ligating substrates, while the ligase with the least bias showed the smallest difference.

While for T4 DNA ligase, T3 DNA ligase, PBCV-1 ligase and hLig3, the majority of correctly base-paired ligation partners were observed in a similar overall frequency, overhangs with the sequence TNNA were ligated inefficiently and notably reduced compared to the average (Supplementary Figure S2). The corresponding ANNT overhangs, despite being expected to be present in the same proportion of the substrate pool, did not show a reduced incidence compared to the other overhangs in the set. We have observed a similar trend previously with three-base overhang ligation by T4 DNA ligase, where TNA sequences were found to be ligated inefficiently, and ligated at a lower rate than other sequences (30). Based on our new data that extends this trend to all tested ligases, we suggest a fundamental inefficiency in ligation of overhang pairs which both contain a 5′-T. Interestingly, an additional subset of inefficiently ligated overhang sequences was observed for hLig3 where overhang pairs which both contain a 5′-C were ligated with greatly reduced efficiency (Supplementary Figure S2C). These sequence preferences are different than those observed previously in nick ligation, and further investigation, including crystallization and structural studies, will be necessary to determine if this inefficiency reflects any differences in the binding or ligation mechanism for pyrimidine-terminated sequences.

### Both degree and type of observed mismatch ligation vary widely by ligase

**Figure 3.**
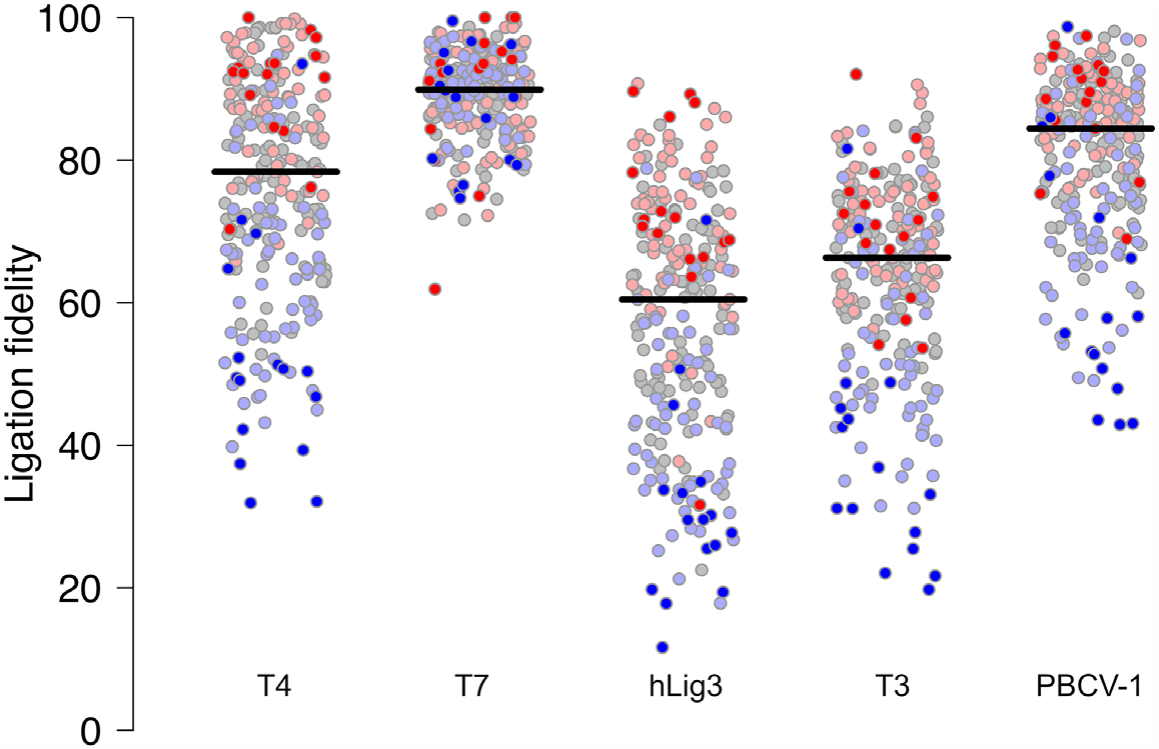
Ligation fidelity for T4 DNA ligase, T7 DNA ligase, hLig3, T3 DNA ligase and PBCV-1 DNA ligase. The fidelity for each of the 256 four-base overhangs was generated by SMRT sequencing of ligation reactions with 100 nM of the multiplexed four-base overhang substrate and 1.75 μM T4 DNA ligase, T7 DNA ligase, hLig3, T3 DNA ligase, or PBCV-1 DNA ligase incubated 1h at 25°C in standard ligation buffer (Supplementary Figures S2A-S2E and File S1 for raw data). Ligation fidelity is defined as the percentage of correct (Watson–Crick) versus incorrect (mismatch) ligation events. A horizontal line indicates the median ligation fidelity of all overhangs for a particular ligase. Each overhang is colored according to its GC content (0% – dark red, 25% – light red, 50% – gray, 75% – light blue, 100% – dark blue).

Ligation fidelity, defined as the fraction of ligation events that are correct (Watson–Crick ligation products) versus incorrect (mismatch products), is summarized in Figure 3. The ligases examined here show extremes of fidelity; while T7 DNA ligase is high fidelity (89% correct ligation products), hLig3 is least faithful (56% correct ligation products), and T4 DNA ligase displays moderate fidelity (72% correct ligation products) (Supplementary Table S2). T4 DNA ligase, T3 DNA ligase, PBCV-1 ligase and hLig3 have a broad range of fidelity for individual overhang sequences, with some overhangs having very few mismatch ligation events and others with frequent mismatch ligations (Figure 3). For many overhangs, even when presented with all possible partners, ligation products are almost exclusively with the Watson Crick partner. For example, when T4 DNA ligase is presented with all possible ligation partners, several overhangs pair with their Watson Crick partner in over 90% of ligation products (e.g. AAAA, AAGA, ACAA, GAAA). However, other overhangs ligate to a partner containing at least one mismatch; in the case of T4 DNA Ligase, several overhangs pair with a mismatch-containing partner more than 60% of the time (e.g. GGCG, GGCC, GGGC, GGGG). Similarly, hLig3, shows a broad range of ligation fidelity: while most overhangs ligate with < 50% fidelity, hLig3 ligates several overhangs (TAAG, AATA, TTAC, CCAA) with > 80% fidelity. In contrast, T7 DNA ligase has a tighter range of ligation fidelity, with only a handful of overhangs that ligate with less than 80% fidelity.

Ligation fidelity generally decreases with increasing GC content of the overhang sequence across most ligases tested (Supplementary Figure S4). However, the strength of this trend depends on the ligase, and the results of significance testing are shown in Supplementary Table S5. For example, T4 DNA ligase, which has an overall fidelity of 72%, has an average fidelity of 90% for overhangs with 0% GC content and decreases in average fidelity with each incremental increase in GC content, ultimately falling to 52% fidelity for overhangs with 100% GC content (Supplementary Figure S4A). The influence of GC content is weaker for the lowest fidelity ligase tested, hLig3, which has an average fidelity of 72% on overhangs with 0% GC content and an average fidelity of 32% for overhangs with 100% GC content (Supplementary Figure S4C). Conversely, the trend is completely absent for T7 DNA ligase, which shows over 86% average fidelity regardless of GC content (Supplementary Figure S4B). Importantly, for all ligases tested, the range of fidelity within each GC content category is sufficiently broad that many instances of individual overhang sequences that break this pattern can be identified.

**Figure 4.**
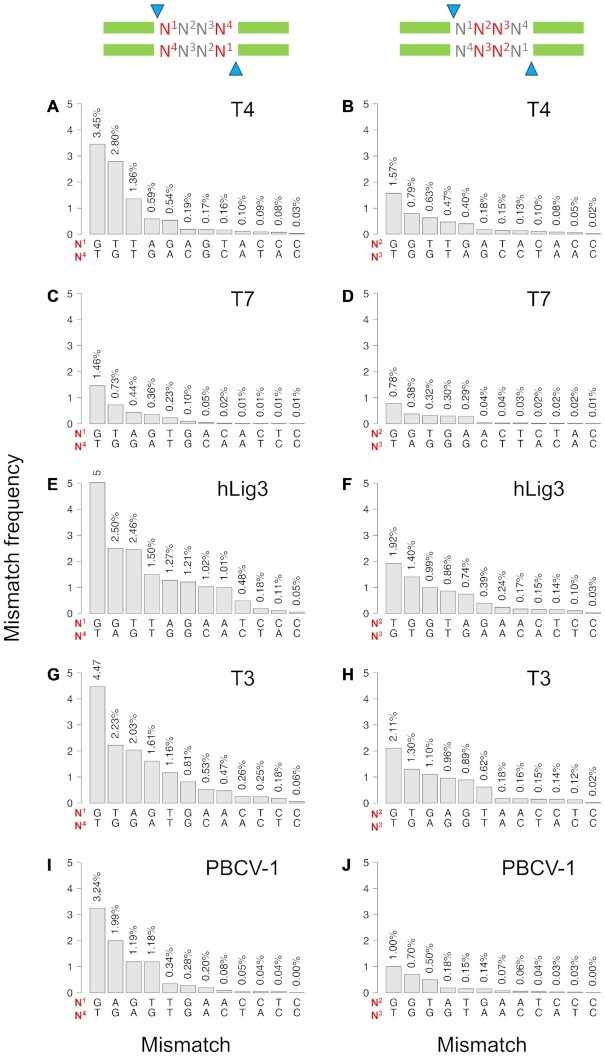
Positional mismatch profiles. The frequency of specific base pair mismatches by position was observed for ligation of four-base overhangs. The results shown are for SMRT sequencing of ligation reactions with 100 nM of the multiplexed four-base overhang substrate and 1.75 μM T4 DNA ligase, T7 DNA ligase, hLig3, T3 DNA ligase, or PBCV-1 DNA ligase incubated 1 h at 25°C in standard ligation buffer (Supplementary Figures S2A–E and File S1 for raw data). (A, C, E, G and I) show the results for the edge position (N1:N4); (B, D, F, H, and J) show the results for the middle position (N2:N3). The overhang positions (N^1^, N^2^, N^3^, N^4^) are numbered from 5′- to 3′- for each strand. Each position in N^1^:N^4^ and N^2^:N^3^ refers to bases in opposite strands. Note that as strand designation is arbitrary, all ligation products were counted in both orientations (top-to-bottom and bottom-to-top strand).

Analysis of the multiplex ligation assay also illuminates the specific mismatch base pairs tolerated by each ligase at the 5′ terminal nucleotide (‘edge’) or base pairs in the middle of the overhang (‘middle’) (Figure 4). Some frequent mismatches, notably G:T mismatches, are common among all tested ligases; however, there are also distinct mismatch pairings observed among the ligation products of each ligase. For T4 DNA ligase, 28% of all ligation products contain a mismatch, and of those mismatch ligation products, 98% have only a single mismatch. Mismatch ligation at the edge position (N1) is dominated by G:T and T:G mismatches, which account for 65% of all mismatch ligations at the edge (Figure 4A). The presence of a mismatch at middle positions (N2 and N3) of the overhang is less tolerated by T4 DNA ligase, but is still dominated by G:T mismatches (Figure 4B). T7 DNA ligase has an overall lower tolerance for mismatch ligation, and only 12% of ligation products contain a mismatch. Similar to T4 DNA ligase, single base pair mismatches account for nearly all (98%) T7 DNA ligase mismatch ligation products and the predominate mismatches are G:T and T:G at the edge position and G:T in the middle position (Figure 4C, D). hLig3 provides a contrast to other ligases, being far more permissive of mismatch pairings both in frequency and in kind. Nearly half of ligation products (44%) contain mismatch base pairs. Interestingly, hLig3 has a significant accumulation of mismatch products with more than a single base pair mismatch, and 8% of ligation products contain two mismatches. Of these double mismatches, the vast majority (97%) involve at least one mismatch in the edge position and typically include at least one G:T mismatch. In addition, while G:T and T:G mismatches are again well tolerated, hLig3, T3 DNA ligase and PBCV-1 ligase are also more permissive of purine:purine mismatches at both the edge and middle positions, with G:A and G:G mismatches ligated almost as frequently as G:T mismatches.

### Addition of ligation enhancer PEG reduces ligation bias but also reduces fidelity

**Figure 5.**
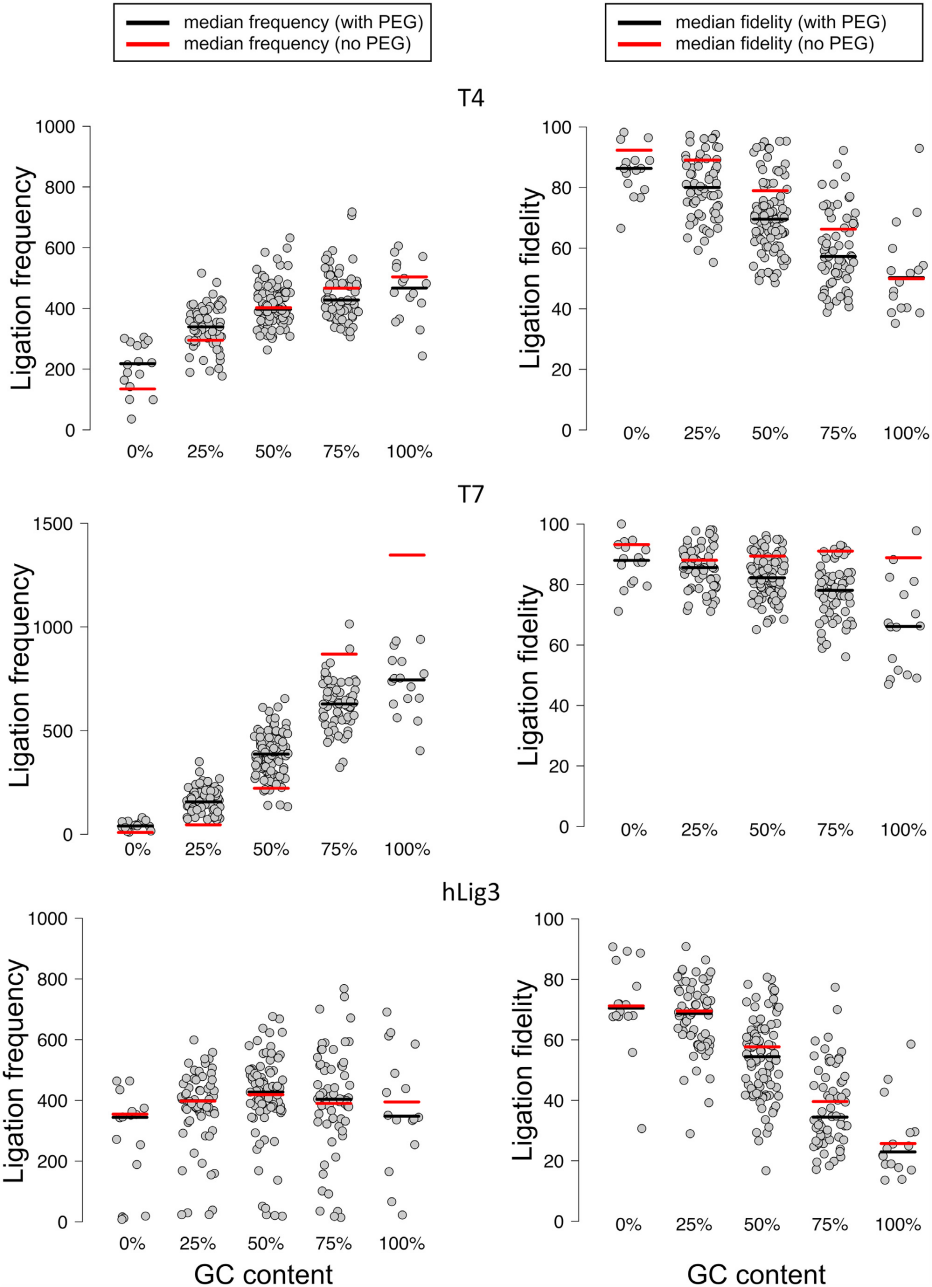
Effect of PEG on ligation fidelity and bias for T4 DNA ligase, T7 DNA Ligase, and hLig3. The normalized ligation frequency and individual overhang fidelity was generated by SMRT sequencing of ligation reactions with 100 nM of the multiplexed four-base overhang substrate and 1.75 μM T4 DNA ligase, T7 DNA ligase or hLig3 incubated 1 h at 25°C in NEBNext^®^ Quick Ligation Buffer (Supplementary Figures S2F–H and File S1 for raw data).The ligation frequency and ligation fidelity of overhangs are grouped by GC content. For comparison to data in standard ligation buffer, the median value is indicated by a horizontal line (red for reactions in standard ligation buffer; black for reactions in PEG-containing buffer).

As PEG is commonly added to stimulate ligation in molecular biology applications, we tested the impact of PEG on end-joining fidelity and bias (32). While the addition of PEG increased the overall library yield for both T4 DNA ligase and T7 DNA ligase (from 61% to 73% and from 20% to 45%, respectively), there was a slight decrease in the yield of hLig3 (from 77% to 72%) (Supplementary Table S2). We found that addition of PEG moderately decreased the overall fidelity of the multiplex ligation reaction for T4 DNA ligase from 72% correct ligation events in the absence of PEG to 67% in the presence of PEG (Supplementary Table S2, Figure 5). The addition of PEG decreased fidelity by the same amount regardless of GC content, except for overhangs with 100% GC content which did not see a change in average fidelity. Interestingly, the fidelity of T7 DNA ligase also significantly decreased from 89% to 78% in the presence of PEG and the overall ligation fidelity of hLig3 had a small decrease upon addition of PEG (56% and 51%, respectively) (Supplementary Table S2, Figure 5). Notably, the addition of PEG did not change the identity of the specific mismatches tolerated for any of the ligases tested (Supplementary Figure S5). Addition of PEG also produced modest changes in the bias of T4 DNA ligase, demonstrated by an overall tightening of the deviation from the average observed ligation frequencies and increased ligation efficiency of some previously inefficient overhangs (Figure 5). In particular, we observed a boost in efficiency for the ligation of overhangs with less than 50% GC content. Interestingly, the extreme bias of T7 DNA ligase is also somewhat remedied by the addition of PEG, and overhangs which were previously not ligated at all have significant product accumulation (Figure 5).

## DISCUSSION

Here, we have completed a comprehensive characterization of the fidelity and bias of DNA ligases that catalyze efficient end-joining *in vitro*. We examined a spectrum of DNA ligases to reveal intriguing differences between end-joining ligation outcomes which suggest contributions from intrinsic structural differences and potential mechanistic differences. By looking at the results of our multiplex assay through the lens of GC content, we determined a variable influence of substrate hydrogen bonding potential on the sequence bias of different DNA ligases. This observation inspired additional experiments measuring the initial velocity rate of ligation on oligo substrates with defined overhang sequences of varying GC content. For some ligases, sequence bias is influenced by annealing equilibria, while other ligases may be less dependent on the strength of overhang annealing due to kinetic or mechanistic factors, such as extremely tight substrate binding. For example, GC content, which corresponds with the Δ*G* of overhang annealing, can account for the extreme bias observed for T7 DNA ligase. This result was observed in both the multiplex assay and in the defined oligo assay, in which the initial velocity of ligation was 1000-fold faster for an overhang with 100% GC content than an overhang with 0% GC content. We therefore suggest that for T7 DNA ligase, the substrate for end-joining ligation is likely exclusively the annealed duplex, with substrates that are predicted to anneal more strongly present in higher concentration and more likely to remain annealed long enough for chemistry to complete. Conversely, ligation efficiency by hLig3 shows only a minimal dependence on GC content. This trend was shown in the multiplex assay and was quantitatively reflected in the defined oligo assay as a more modest 10-fold difference in the initial velocity rate between the fastest (75% GC content) and slowest (0% GC content) overhangs. The differences in both broad sequence bias trends and kinetic rates of the enzymes may be suggestive of differing mechanisms for end-joining. We note that no abortive adenylated intermediate (AppDNA) was observed in the defined oligo experiments with any of the ligases tested. Typically, a buildup of AppDNA is observed for poorly ligated substrates that fall out of the active site after transfer of the adenyl group but before nick sealing is completed. The lack of AppDNA is suggestive that for cohesive end-joining, the substrate bound by the DNA ligase is the annealed substrate. However, previous work suggests that the binding domains of hLig3 can independently bind two different DNA ends, bringing them into close proximity and potentially stimulating annealing due to effective concentration effects and preventing dissociation of one end prior to chemistry (11). Alternatively, the bias may be due to dissociation rates, with high fidelity DNA ligases like T7 frequently dissociating from weakly annealed ends before the chemical steps can take place, and low bias ligases like hLig3 able to bind long enough to permit joining even for poorly paired substrates. In the current work, we cannot rule out a stepwise binding of the two ends with strong annealing serving to stabilize the structure long enough to complete ligation. Perhaps, therefore, these data suggest that some enzymes may utilize a mixture of mechanisms for end-joining.

In general, the differences in sequence preference between the viral ligases at least partially derive from varied influence of substrate GC content. However, our analysis also revealed that beyond the influence of overhang GC content, there are additional sequence biases specific to each enzyme. In addition, the sequence preferences of hLig3 are completely unlike the viral ligases. We have previously suggested that differences in the ability of DNA ligases to join substrates with different end structures (i.e. cohesive ends versus blunt ends vs. single base overhangs) are not solely due to the strength of DNA binding or the presence of DNA binding domains (29). The new insight gained here suggests that there may be enzyme-specific contributions to sequence specificity as well. While there are some known active site structural distortions and constraints which could influence ligation efficiency, such as the ability of the ligase to distort the DNA helix, this has not been fully explored, particularly in the context of end-joining (33). There are currently no co-crystal structures with end-joining substrates available for the ligases examined here, but structures of T4 DNA ligase, PBCV-1 DNA ligase and hLig3 bound to nick DNA substrates are available, as well as an apo structure of T7 DNA ligase. It is worth noting that with exception of T3 and T7 DNA ligases, which share 68% sequence identity, all other ligases studied here have quite divergent sequences (less than 20%, Supplementary Table S6). In order to compare enzyme-specific contributions to sequence bias, we constructed an overlay of the available DNA ligase crystal structures. Upon examination of the published crystal structures, we note that despite the lack of sequence identity and differences in the overall sizes and domains of these enzymes, the active sites of these ligases align remarkably well, with only conservative differences in amino acid identities (Supplementary Figure S6A and S6B). A high degree of structural conservation of the nucleotidyl transferase domain among DNA ligases has been previously described (34). However, the alignments showed marked differences in the DNA binding regions derived from the interface of multiple domains, with major differences in the ligase contacts with the DNA phosphodiester backbone (Supplementary Figure S6C). For example, there are 28, 19 and 20 amino acid residues within 3.6 Å of the phosphodiester backbone in the T4 DNA ligase, PBCV-1 DNA ligase, and hLig3 DNA co-crystal structures, respectively. Of these residues, only five residues were in similar positions in all three ligases, most of which were proximal to the active site. The T7 DNA ligase structure, which lacks DNA, includes seven of the DNA contacting residues found in at least one of the other ligases. Of these, three were shared between all four ligases. Interestingly, Ser407 in T4 DNA ligase and Thr249 in PBCV-1 DNA ligase are the only residues that appear to contact DNA bases rather than the phosphodiester backbone (6,7). These residues align very well and lack a direct DNA base-interacting counterpart in hLig3, where Gly644 is in the same position. Mutation of Thr249 in PBCV-1 retained function on a nick substrate, but mutation of the corresponding residue has not been tested in other ligases or with an end-joining substrate and is therefore a potential residue for further exploration in structural studies (6). Further, hLig3 is the only ligase examined which contains a Zn-finger DNA binding domain, and the presence or absence of this unique DNA binding domain may also influence the sequence preferences of the ligase. Additional studies, such as co-crystal structures with end-joining substrates containing different sequences of interest identified here, may clarify these observed differences in specificity.

Our previous work examining three- and four-base overhang ligation of T4 DNA Ligase uncovered a bias against TNA and TNNA sequences and determined that this inefficiency was due to dramatically lower ligation rates of these sequences. Here we extended this observation to all DNA ligases examined, which all showed a particular inefficiency in ligating overhangs with the sequence TNNA. In the work presented here, we also observed an additional specific bias against CNNG sequences for hLig3. Additional follow up studies to this work will relate to the observed bias against 5′ pyrimidines. As the sequence preferences observed here are different than those observed previously in nick ligation, further investigation, including co-crystallization with TNNA substrates and kinetic studies, will help define the basis for this inefficiency, whether enzyme-substrate binding or the ligation mechanism for pyrimidine-terminated sequences.

Our data also reveal a complex relationship between ligase identity and fidelity. While the GC content of the overhang plays a role, the ligase-specific observations highlight that properties intrinsic to the enzyme have a significant influence on ligase fidelity. It is likely that some ligases are more tolerant of mismatches due to a more flexible active site, and additional factors may also contribute, such as enforcement of proper geometry for high fidelity ligation by metal binding sites (35). The information gained here on mismatch tolerance of different DNA ligases in end-joining will inform future structure-function studies. Further, the complexity of trends observed emphasizes the importance of careful selection of both overhang sequences and DNA ligase based on empirical data, rather than broad trends or ‘rules of thumb’ in applications such as Golden Gate cloning where accurate and efficient ligation is required. Surprisingly, mismatches proximal to the site of ligation are better tolerated than mismatches in the middle of the four-base overhang. Thus, special attention must be paid to the potential for mismatch ligation at this position when choosing overhangs. Ultimately, fidelity is dependent on availability of mispairing partners, and in overhang subsets lacking any high efficiency mismatch partners, even the lowest fidelity overhang can ligate exclusively to its Watson–Crick partner if all high frequency mismatch partners are missing from the set.

Our work on end-joining ligation fidelity supplements previous studies on ligation in the context of mismatch-containing nicked DNA (18–20,27,36), and finds the following similarities and differences. As in nick ligation, G:T mismatches were the most frequently observed mismatches for all tested ligases in cohesive end-joining. This is perhaps not surprising, as the G:T mismatch forms two hydrogen bonds and fits within the standard DNA helix width with minimal perturbation (37–39). Indeed, G:T mismatches are also frequent mismatch incorporation errors by polymerases. It therefore makes sense that there are cellular enzymatic pathways which specifically recognize and remove this common replication and repair error, such as mismatch repair, base excision repair by DNA glycosylases, and other mismatch endonucleases (40–42). Potentially more interesting is our finding that in the context of end-joining ligation, some DNA ligases, including hLig3, PBCV-1 ligase and T3 DNA ligase are able to accommodate the helix distorting purine:purine mismatches, which are larger and more dynamic than pyrimidine:pyrimidine or pyrimidine:purine mismatches (37). While we would perhaps expect that a very tight binding enzyme like hLig3 might overcome deviations in substrate structure to make all mismatches more permissible, it is interesting that only some mismatches are greatly enhanced, suggesting a role for both active site flexibility and simple tight binding in mismatch tolerance. Further investigation to determine if mismatches are more permitted during ligation of the first strand break rather than second ligation event during an end-joining ligation may reveal additional substrate flexibility during the end-joining mechanism.

The properties of DNA ligases as revealed by this study inform ligase choice and highlight that some ligases are better suited to certain applications than others. For example, it is clear that T4 DNA ligase has been useful for complex assemblies because of its balance between relatively low bias, allowing for the inclusion of many efficient overhangs, and fidelity that is mostly constrained to G:T mismatches. It is also possible to imagine applications where a low fidelity ligase like hLig3 may prove especially desirable. For example, chromosome conformation capture techniques investigate folded chromatin structures to determine spatially close regions of the genome. These techniques rely on digestion of the genome followed by re-ligation, and involve complicated secondary structures as well as the need for high efficiency end-joining ligation (43). As hLig3 performs well when ligating a mixed pool of overhangs, both in overall yield and ability to ligate a variety of mismatches, it may prove useful for this type of application. Given the complexity of these fidelity trends, the data sets for all ligases examined here have been added to our previously reported webtool to allow users to examine how the choice of ligase and overhang sequence are likely to impact ligation outcomes (44).

These data further inform how typical reaction conditions and additives may impact different DNA ligases and provide insight on modifications that might improve particular application outcomes. For example, for applications such as cloning or adaptor ligation, the boost in ligation product yield from adding PEG will likely outweigh the moderate loss of fidelity for T4 DNA Ligase and T7 DNA Ligase. However, for applications involving highly complex multi-fragment assembly, the loss of fidelity observed when adding PEG may require more consideration of the particular overhangs used to limit potential mismatch ligation among an overhang set. For T7 DNA Ligase, the addition of PEG may make the enzyme a more attractive candidate for applications requiring many fragments, as gains in efficiency for additional overhangs will expand the pool of efficient potential overhang sequences, while the small loss in fidelity is tolerable due to the high overall fidelity of this enzyme. These trends may reflect the mechanism of PEG in ligation enhancement, which works via a volume exclusion effect to increase the effective concentration of substrate and enzyme (45,46). The resulting macromolecular crowding ultimately favors protein binding, which we suggest is more crucial to the activity of T7 DNA ligase.

One important future direction for this work is the examination of DNA ligase fidelity and sequence bias on different end structures. While we have currently focused on 5′ four base overhangs, all ligases studied here can also join substrates with 3′ overhangs. In addition, many of the ligases examined are also capable of single base overhang and blunt end-joining. The surprising complexity of the trends discovered here suggests that the observations and trends on 5′ overhang structures cannot simply be extended to different overhang lengths or polarities. In addition, in this work we have only examined the sequence context on the phosphate donor strand. Previous studies using nicked DNA substrates have indicated that most ligases are less tolerant of mismatches at the 3′ side (hydroxyl acceptor) of the ligation junction, and we are curious to see if these observations extend to end-joining ligation as well. Additional substrate design strategies will be required to carry out these experiments, with careful selection of restriction enzymes to enable a variety of end lengths and structures as well as randomized regions close to the ligation junction.

In this work, we extended our original high-throughput, sequencing based methodology to determine the sequence bias and fidelity of cohesive end ligation for a range of DNA ligases. Our findings provide novel insight into the complex biology of end-joining reactions and the differences between individual DNA ligases. We have identified key mismatch and bias events that will be targeted in depth in future structure–function and mechanistic studies on DNA ligase end-joining activity. Finally, the insight gained can also be applied to develop and optimize new ligation-based workflows for biotechnology applications.

## DATA AVAILABILITY

Raw ligation product observation counts were provided as CSV formatted data tables in Supplementary Data. Custom software tools are available in the GitHub repository at: https://github.com/potapovneb/ligase-fidelity.

## Supplementary Material

gkac241_Supplemental_Files
