# Supplementary material for: Mismatch discrimination and sequence bias during end-joining by DNA ligases
Source: Nucleic Acids Res. 2022 Apr 19;50(8):4647–58. doi: 10.1093/nar/gkac241 (PMC9071435; doi:10.1093/nar/gkac241)
Supplement: gkac241_Supplemental_Files [file gkac241_supplemental_files.zip › Supplementary Data_NAR clean revision.pdf]

SUPPLEMENTARY DATA

Table S1. Ligation substrate and ligation library sequences

Substrate	Sequence [1]
Precursor oligonucleotide	TCACGTNNNNG**GAGACC**TGCGATCCAGTGCGCCGTCCATTGATCAACGNNNNNNCAAATCTCTCTCTTTTCCTCCTCCTCCGTTGTTGTTGTTGAGAGAG
Ligation profile substrate	pNNNNG**GAGACC**TGCGATCCAGTGCGCCGTCCATTGATCAACGNNNNNNCAAATCTCTCTCTTTTCCTCCTCCTCCGTTGTTGTTGTTGAGAGAGATTTGNNNNNNCGTTGATCAATGGACGGCGCACTGGATCGCA**GGTCTC**C
Expected insert [2]	TTGNNNNNNCGTTGATCAATGGACGGCGCACTGGATCGCAGGTCTCCNNNNGGAGACCTGCGATCCAGTGCGCCGTCCATTGATCAACGNNNNNNCAA

[1] The type IIS restriction enzyme recognition site is indicated in bold. SMRT adapter region is underlined.

[2] The expected insert length is 99nt. The location of four-base overhang is in position 48..51, 3′-randomized region is in position 4..9, and 5′-randomized region is position 90..95.

Table S2. Summary of ligation fidelity and bias experiments

Ligase	Total ligations	Correct ligations	Mismatch ligations	Overall fidelity, % [a]	Yield, %
T4 DNA ligase	158,614	114,783	43,831	72.4 ± 0.6	61 ± 1.4
T7 DNA ligase	169,136	150,212	18,924	88.8 ± 0.2	20 ± 0.1
hLig3	321,746	180,316	141,430	55.9 ± 1.6	77 ± 1.5
T3 DNA ligase	172,210	102,085	70,125	59.2 ± 1.6	55 ± 1.5
PBCV-1 ligase	113,923	87,936	25,987	77.4 ± 0.6	50 ± 0.5
T4 DNA ligase (PEG)	209,092	139,890	69,202	66.9 ± 0.7	73 ± 1.6
T7 DNA ligase (PEG)	66,562	51,884	14,678	77.8 ± 0.1	45 ± 0.6
hLig3 (PEG)	187,912	94,525	93,387	51.4 ± 0.0	72 ± 2.5

[a] The overall fidelity was computed by combining all replicate ligation fidelity data. The standard deviation was computed based on overall fidelities per replicate. Two replicates were collected for each ligase with exception of hLig3 with 4 replicates, and T3 DNA ligase with 3 replicates.

Table S3. Defined 5’ four base overhang, FAM-labeled[1] oligo substrate pairs

Substrate	Sequence [1]
0% GC (ATAA/TATT)	5′-pATAACGAAGAGCTGCGATCCAGTGGGCCGTGC(FAM)-3′
	3′-GCTTCTCGACGCTAGGTCACCCGGCACG-5′
	5′-pTTATGATGGGCAGTGGCCGGTAGC(FAM)-3′
	3′-CTACCCGTCACCGGCCATCG-5′
25% GC (CTAT/ATAG)	5′-pCTATCGAAGAGCTGCGATCCAGTGGGCCGTGC(FAM)-3′
	3′-GCTTCTCGACGCTAGGTCACCCGGCACG-5′
	5′-pATAGGATGGGCAGTGGCCGGTAGC(FAM)-3′
	3′-CTACCCGTCACCGGCCATCG-5′
50% GC (GTGA/TCAC)	5′-pGTGACGAAGAGCTGCGATCCAGTGGGCCGTGC(FAM)-3′
	3′-GCTTCTCGACGCTAGGTCACCCGGCACG-5′
	5′-pTCACGATGGGCAGTGGCCGGTAGC(FAM)-3′
	3′-CTACCCGTCACCGGCCATCG-5′
75% GC (AGCG/CGCT)	5′-pAGCGCGAAGAGCTGCGATCCAGTGGGCCGTGC(FAM)-3′
	3′-GCTTCTCGACGCTAGGTCACCCGGCACG-5′
	5′-pCGCTGATGGGCAGTGGCCGGTAGC(FAM)-3′
	3′-CTACCCGTCACCGGCCATCG-5′
100% GC (CGGC/GCCG)	5′-pCGGCCGAAGAGCTGCGATCCAGTGGGCCGTGC(FAM)-3′
	3′-GCTTCTCGACGCTAGGTCACCCGGCACG-5′
	5′-pGCCGGATGGGCAGTGGCCGGTAGC(FAM)-3′
	3′-CTACCCGTCACCGGCCATCG-5′

[1] FAM stands for 6-carboxyfluorescein

[2] In the substrate descriptions, overhang pairs are written in the 5′ to 3′ direction with the phosphate omitted

Table S4. Initial velocity measurements[a] of DNA ligases with defined 5 four base overhang substrates of varying GC content

Substrate[b]	T4 DNA ligase V_0 (min^{-1})	T7 DNA ligase V_0 (min^{-1})	hLig3 V_0 (min^{-1})
0% GC (ATAA/TATT)	0.16 ± 0.037	0.003 ± 0.0020	0.058 ± 0.013
25% GC (CTAT/ATAG)	0.70 ± 0.068	0.028 ± 0.0026	0.18 ± 0.016
50% GC (GTGA/TCAC)	3.6 ± 0.12	0.13 ± 0.0090	0.24 ± 0.0087
75% GC (AGCG/CGCT)	18 ± 0.62	1.4 ± 0.086	0.61 ± 0.015
100% GC (CGGC/GCCG)	15 ± 1.7	2.2 ± 0.13	0.29 ± 0.0075

[a] The reported values were obtained by fitting a linear regression to the data up to a maximum of 25% product formation. The error represents the reported error in the slope of the fit.

[b] Substrate sequence details are in Table S3.

Table S5. Statistical significance values for testing ligation fidelity difference between overhangs with various GC content [a]

Ligase	0% vs 25% GC	25% vs 50% GC	50% vs 75% GC	75% vs 100% GC
T4	1.4E-01	4.4E-07	4.2E-08	5.4E-04
T7	7.3E-02	7.5E-01	3.1E-02	8.4E-02
hLig3	5.4E-01	3.0E-07	2.1E-09	8.8E-04
T3	8.4E-01	1.3E-02	4.2E-08	8.8E-04
PBCV-1	3.0E-01	1.4E-02	2.8E-05	1.4E-03

[a] The reported values are p-values from two-sample Kolmogorov-Smirnov test.

Table S6. Pairwise sequence identity between ligases

Ligase	T4	T7	hLig3	T3	PBCV-1
**T4**		12%	8%	12%	11%
**T7**	12%		7%	67%	17%
**hLig3**	8%	7%		7%	5%
**T3**	12%	67%	7%		18%
**PBCV-1**	11%	17%	5%	18%	

A Ligation pool

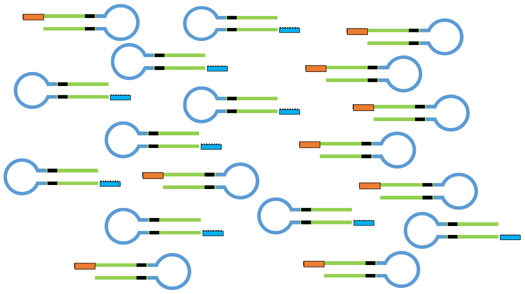

B Ligation products

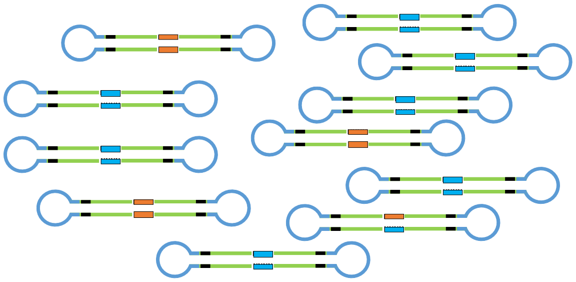

C Fidelity and bias

Overhang	Total	Correct	Mismatch	Fidelity
overhang #1	8	7	1	88%
overhang #2	4	3	1	75%
…				

Figure S1. Ligation fidelity and bias assay schematic. (**A**) Libraries containing randomized four-base overhangs were synthesized. Sample-randomized overhang pairs are schematically illustrated as blue and orange bars. (**B**) Ligation substrates are ligated with DNA ligase, and correct (same overhang color in figure) and mismatch-containing (different overhang color) products are formed. (**C**) Ligation fidelity is defined as the fraction of correct ligations. Ligation bias describes the relative total numbers of ligation products formed for each overhang. For more details on the assay and bioinformatics, see References 30 and 31.

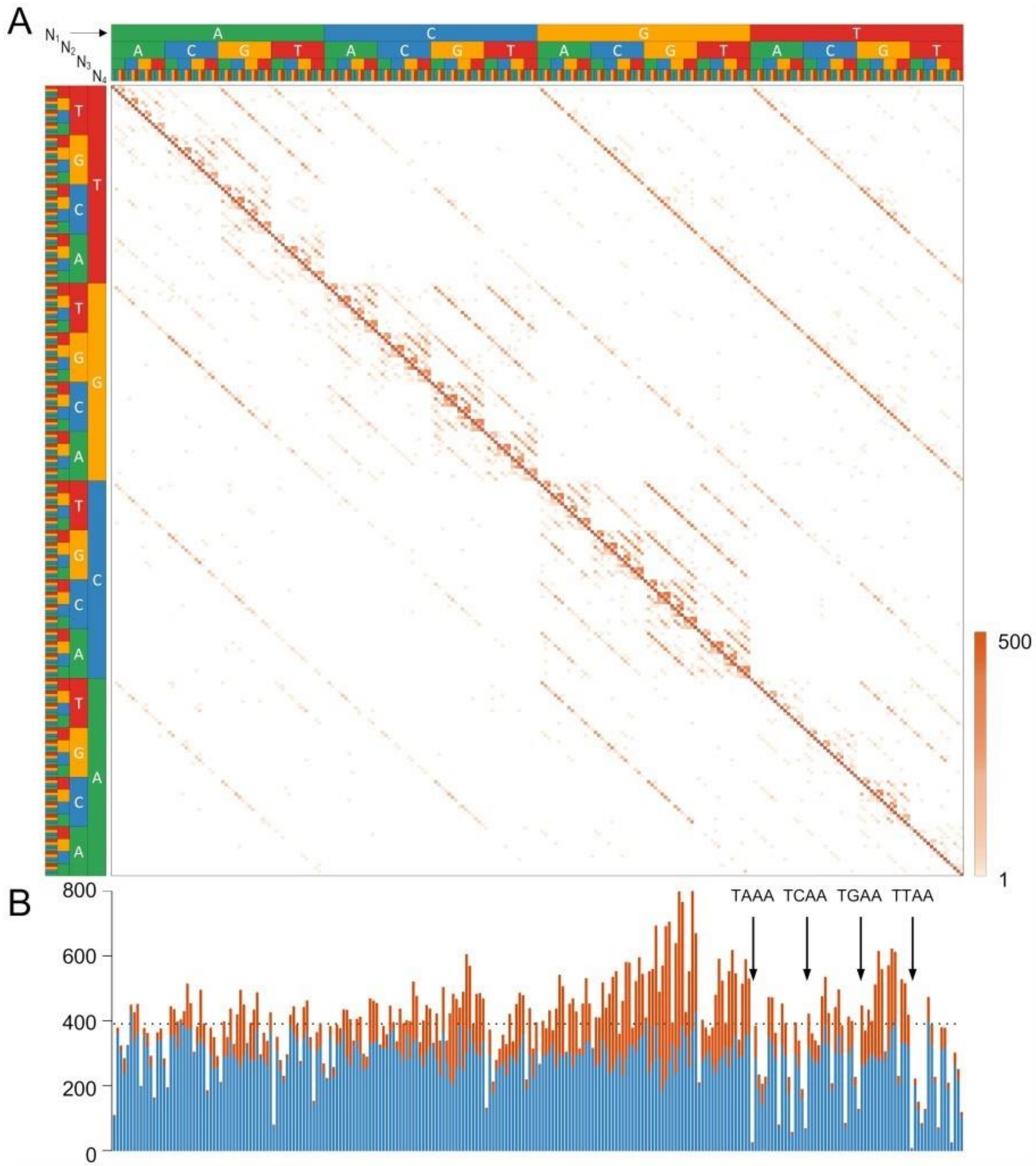

Figure S2A. Multiplex ligation assay results for the ligation of randomized four-base overhangs by T4 DNA ligase. SMRT sequencing results for ligating 100 nM of the multiplexed four-base overhang substrate 1 h at 25°C, with 1.75 µM T4 DNA ligase in standard ligation buffer. Observations have been normalized to 100,000 ligation events (File S1 for raw observation totals). (**A**) Frequency heat map of all ligation events (log-scaled). Overhangs are listed alphabetically left to right (AAAA, AAAC, AAAG …TTTG, TTTT) and bottom to top such that the Watson–Crick pairings are shown on the diagonal. (**B**) Stacked bar plot showing the frequency of ligation products containing each overhang, corresponding to each column in the heat map in (A). Fully Watson–Crick paired ligation results are indicated in blue, and ligation products containing one or more mismatches are in orange. Note: This data previously appeared in Reference 31, Supporting Figure S1.

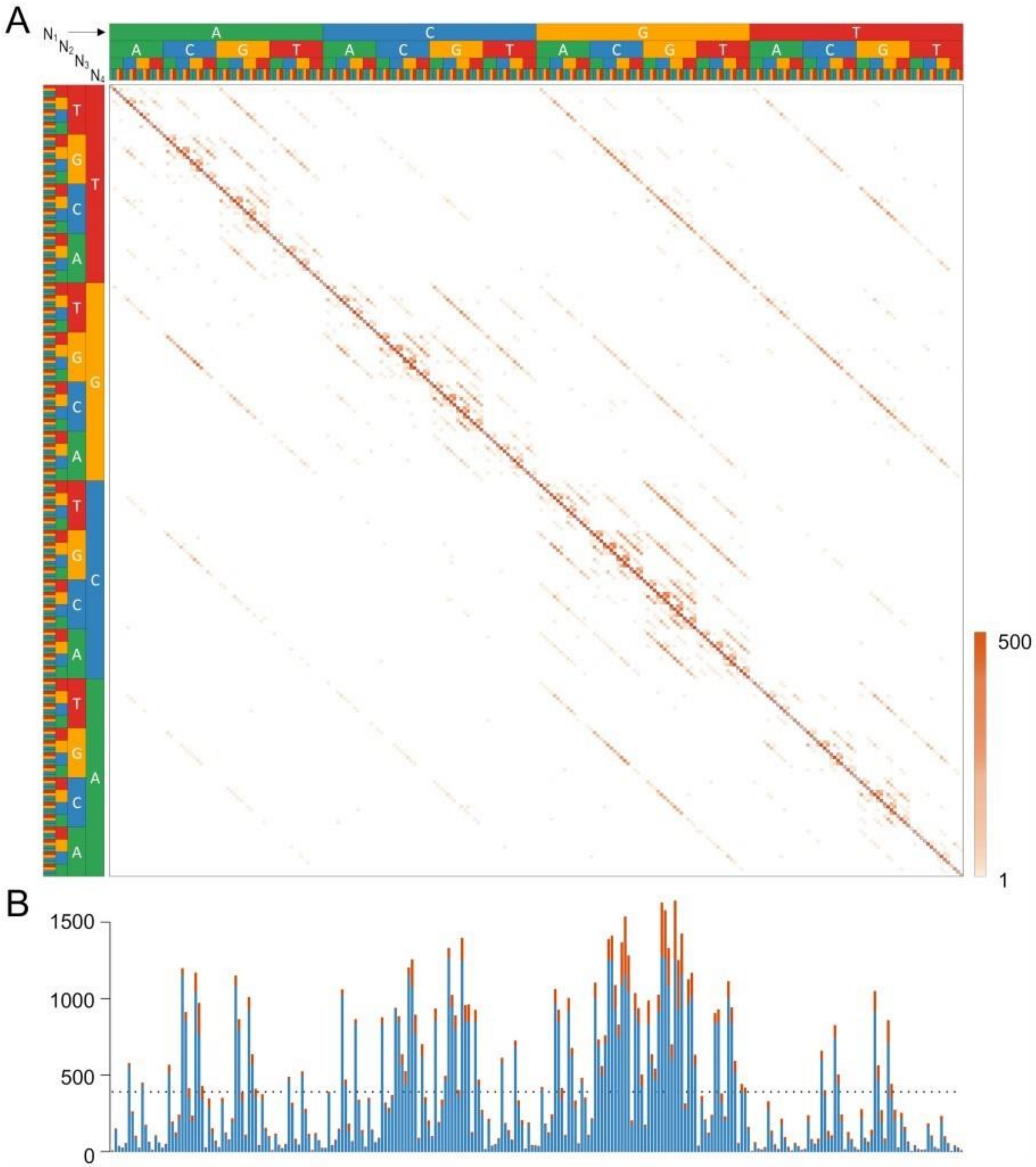

Figure S2B. Multiplex ligation assay results for the ligation of randomized four-base overhangs by T7 DNA ligase. SMRT sequencing results for ligating 100 nM of the multiplexed four-base overhang substrate 1 h at 25°C, with 1.75 µM T7 DNA ligase in standard ligation buffer. Observations have been normalized to 100,000 ligation events (File S1 for raw observation totals). (**A**) Frequency heat map of all ligation events (log-scaled). Overhangs are listed alphabetically left to right (AAAA, AAAC, AAAG …TTTG, TTTT) and bottom to top such that the Watson–Crick pairings are shown on the diagonal. (**B**) Stacked bar plot showing the frequency of ligation products containing each overhang, corresponding to each column in the heat map in (A). Fully Watson–Crick paired ligation results are indicated in blue, and ligation products containing one or more mismatches are in orange.

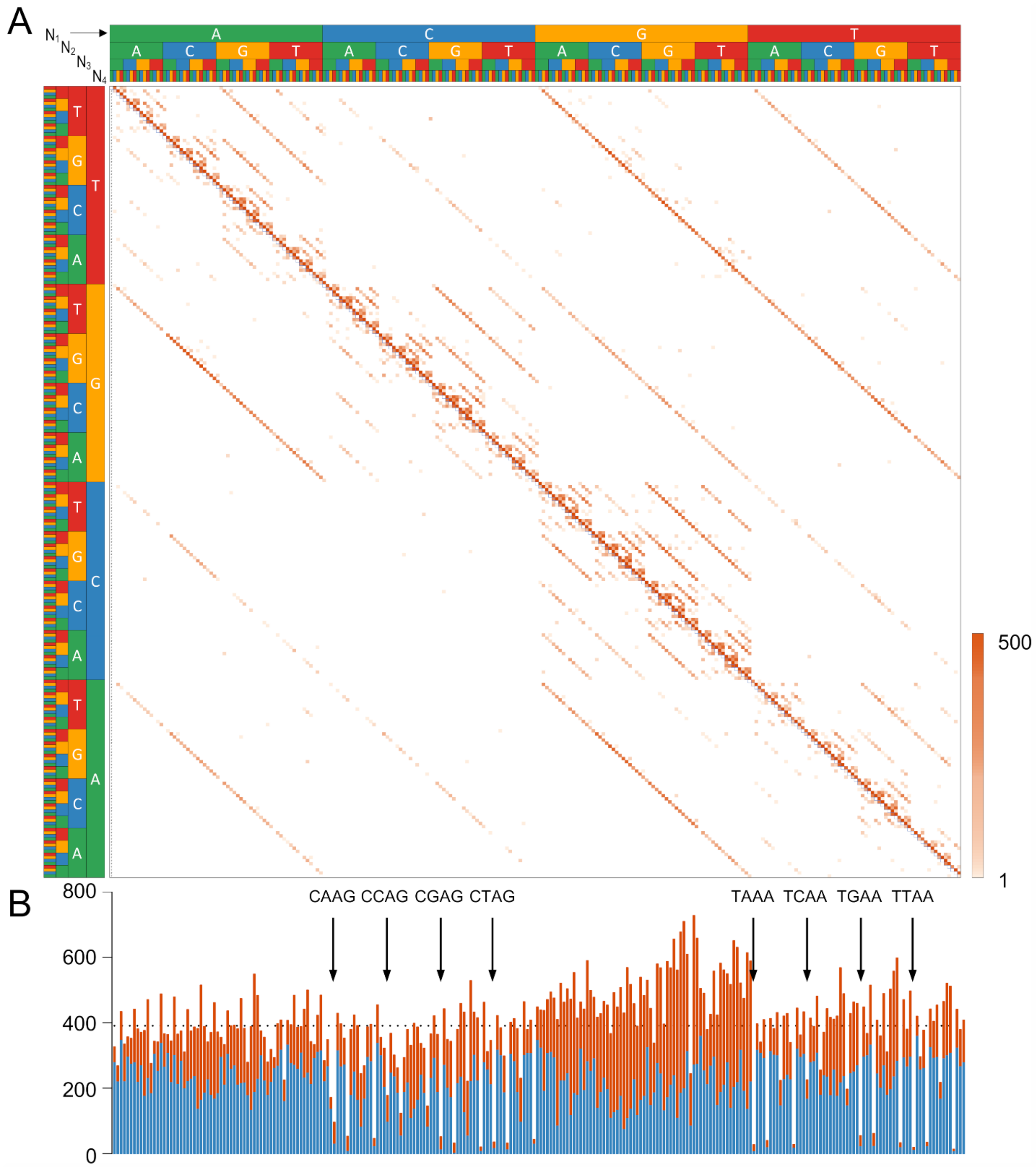

Figure S2C. Multiplex ligation assay results for the ligation of randomized four-base overhangs by hLig3. SMRT sequencing results for ligating 100 nM of the multiplexed four-base overhang substrate 1 h at 25°C, with 1.75 μM hLig3 in standard ligation buffer. Observations have been normalized to 100,000 ligation events (File S1 for raw observation totals). (**A**) Frequency heat map of all ligation events (log-scaled). Overhangs are listed alphabetically left to right (AAAA, AAAC, AAAG …TTTG, TTTT) and bottom to top such that the Watson–Crick pairings are shown on the diagonal. (**B**) Stacked bar plot showing the frequency of ligation products containing each overhang, corresponding to each column in the heat map in (A). Fully Watson–Crick paired ligation results are indicated in blue, and ligation products containing one or more mismatches are in orange.

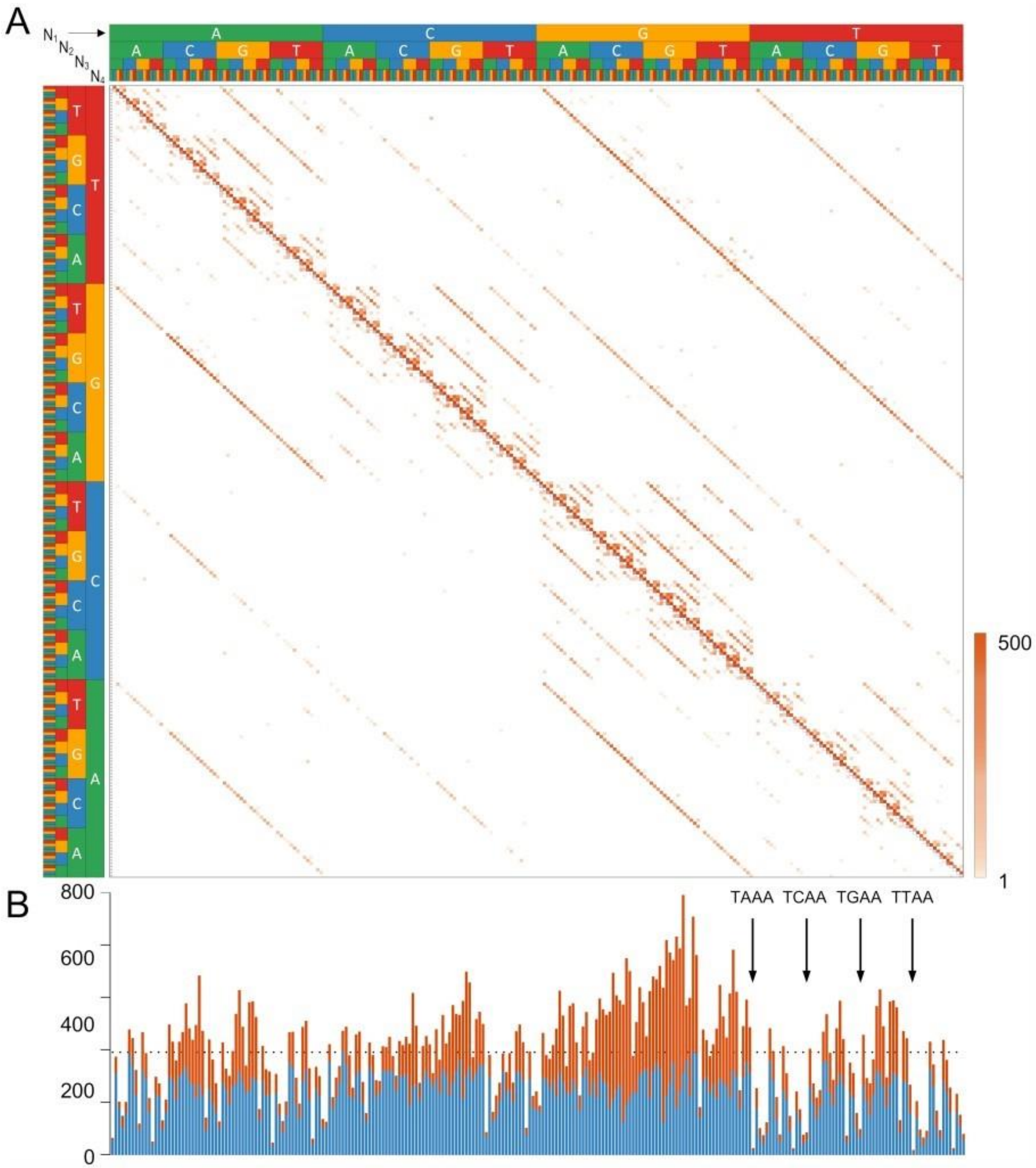

Figure S2D. Multiplex ligation assay results for the ligation of randomized four-base overhangs by T3 DNA ligase. SMRT sequencing results for ligating 100 nM of the multiplexed four-base overhang substrate 1 h at 25°C, with 1.75 μM T3 DNA ligase in standard ligation buffer. Observations have been normalized to 100,000 ligation events (File S1 for raw observation totals). (**A**) Frequency heat map of all ligation events (log-scaled). Overhangs are listed alphabetically left to right (AAAA, AAAC, AAAG …TTTG, TTTT) and bottom to top such that the Watson–Crick pairings are shown on the diagonal. (**B**) Stacked bar plot showing the frequency of ligation products containing each overhang, corresponding to each column in the heat map in (A). Fully Watson–Crick paired ligation results are indicated in blue, and ligation products containing one or more mismatches are in orange.

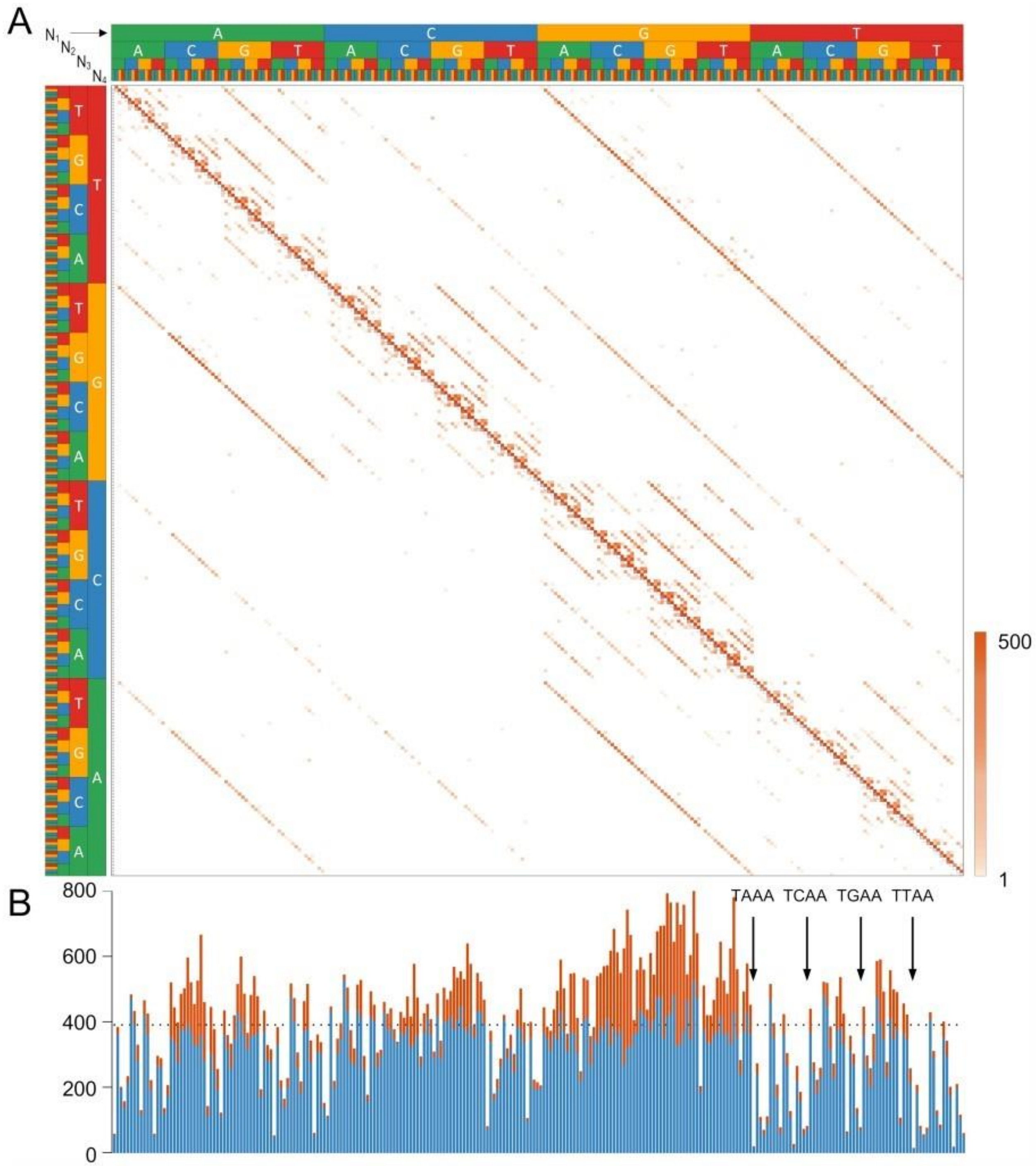

Figure S2E. Multiplex ligation assay results for the ligation of randomized four-base overhangs by PBCV-1 DNA ligase. SMRT sequencing results for ligating 100 nM of the multiplexed four-base overhang substrate 1 h at 25°C, with 1.75 µM PBCV-1 DNA ligase in standard ligation buffer. Observations have been normalized to 100,000 ligation events (File S1 for raw observation totals). (**A**) Frequency heat map of all ligation events (log-scaled). Overhangs are listed alphabetically left to right (AAAA, AAAC, AAAG …TTTG, TTTT) and bottom to top such that the Watson–Crick pairings are shown on the diagonal. (**B**) Stacked bar plot showing the frequency of ligation products containing each overhang, corresponding to each column in the heat map in (A). Fully Watson–Crick paired ligation results are indicated in blue, and ligation products containing one or more mismatches are in orange.

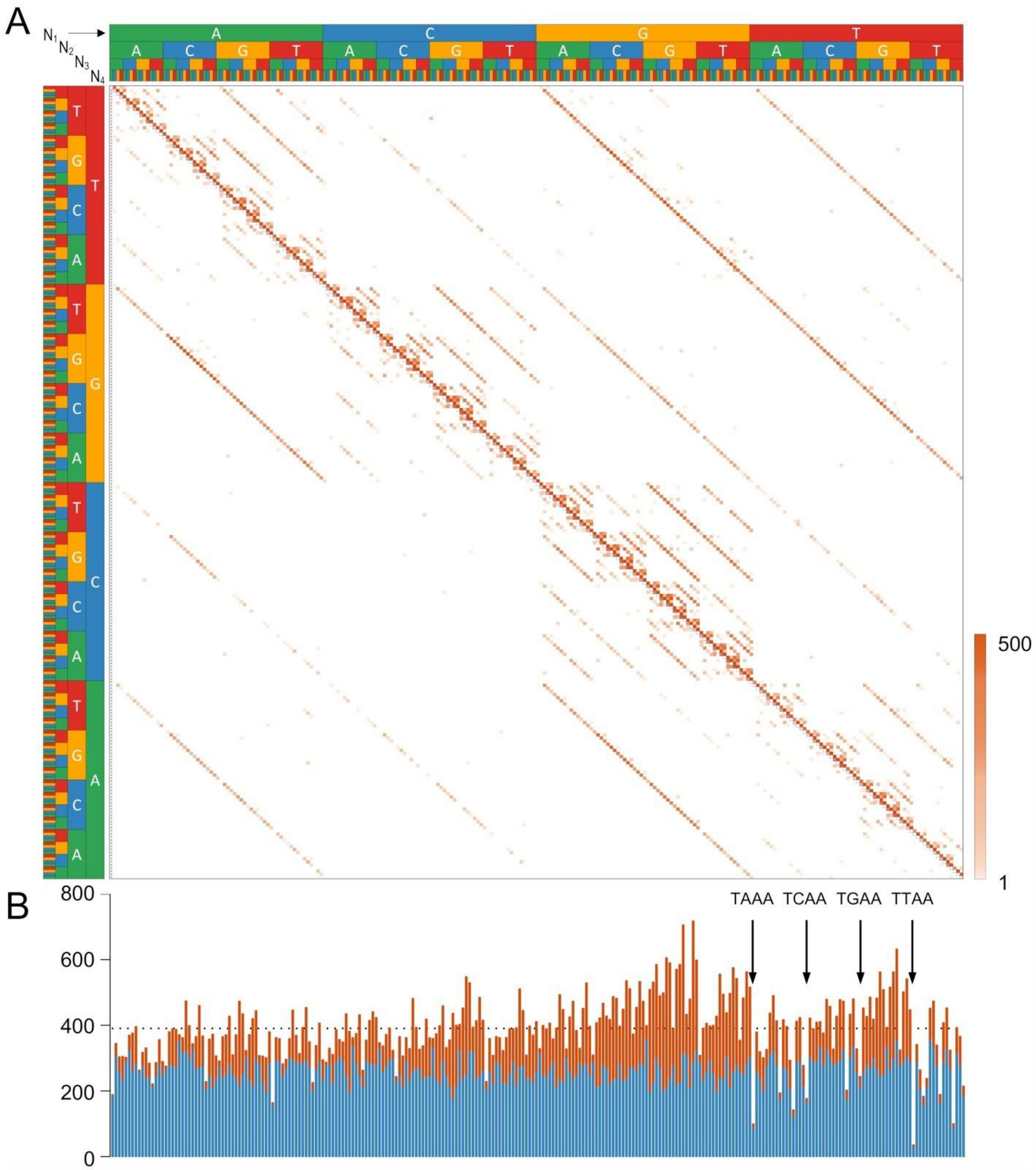

Figure S2F. Multiplex ligation assay results for the ligation of randomized four-base overhangs by T4 DNA ligase (PEG buffer). SMRT sequencing results for ligating 100 nM of the multiplexed four-base overhang substrate 1 h at 25°C, with 1.75 μM T4 DNA ligase in Quick ligation buffer. Observations have been normalized to 100,000 ligation events (File S1 for raw observation totals). (**A**) Frequency heat map of all ligation events (log-scaled). Overhangs are listed alphabetically left to right (AAAA, AAAC, AAAG …TTTG, TTTT) and bottom to top such that the Watson-Crick pairings are shown on the diagonal. (**B**) Stacked bar plot showing the frequency of ligation products containing each overhang, corresponding to each column in the heat map in (A). Fully Watson–Crick paired ligation results are indicated in blue, and ligation products containing one or more mismatches are in orange.

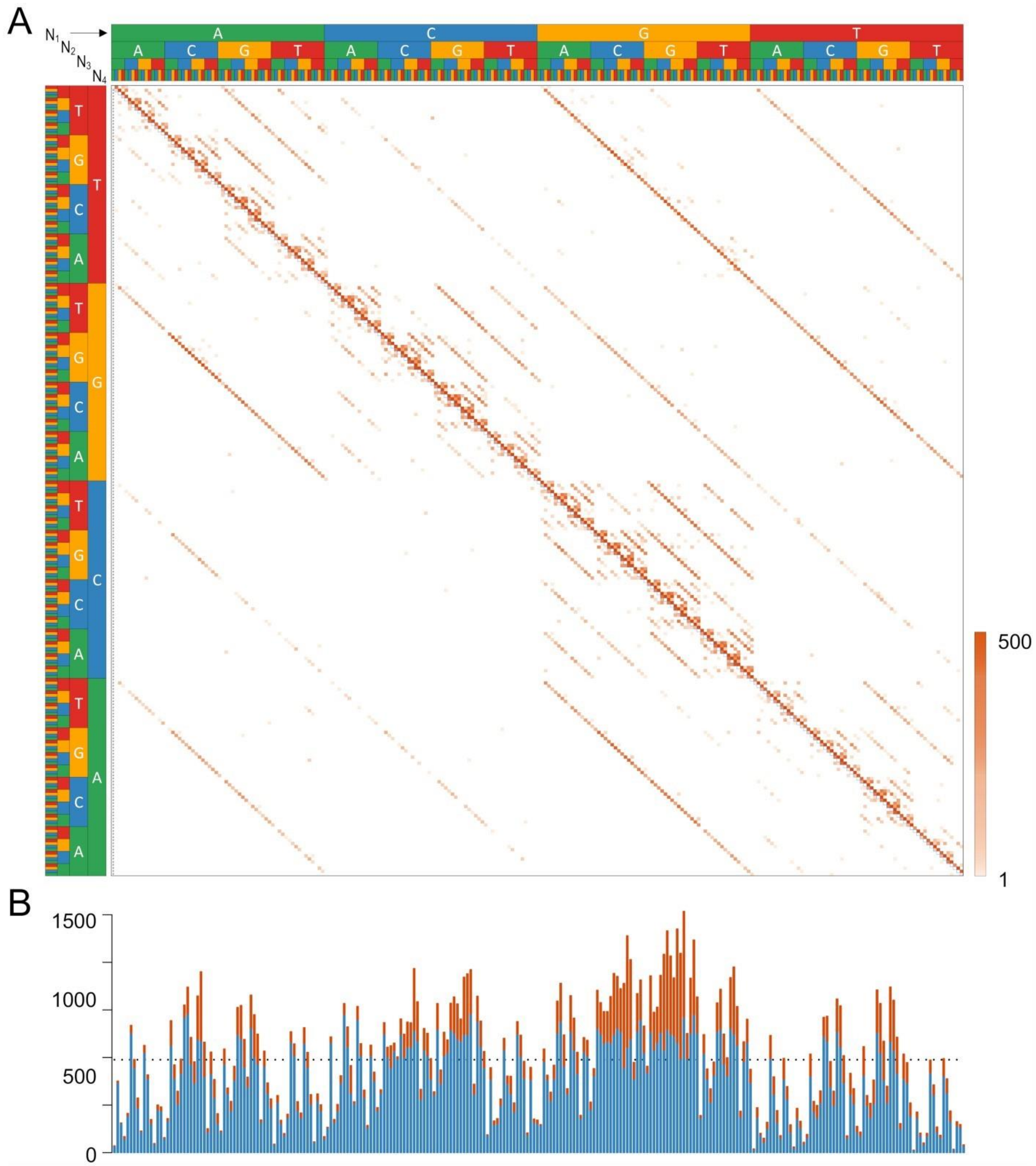

Figure S2G. Multiplex ligation assay results for the ligation of randomized four-base overhangs by T7 DNA ligase (PEG buffer). SMRT sequencing results for ligating 100 nM of the multiplexed four-base overhang substrate 1 h at 25°C, with 1.75 μM T7 DNA ligase in Quick Ligation Buffer. Observations have been normalized to 100,000 ligation events (File S1 for raw observation totals). (**A**) Frequency heat map of all ligation events (log-scaled). Overhangs are listed alphabetically left to right (AAAA, AAAC, AAAG …TTTG, TTTT) and bottom to top such that the Watson–Crick pairings are shown on the diagonal. (**B**) Stacked bar plot showing the frequency of ligation products containing each overhang, corresponding to each column in the heat map in (A). Fully Watson–Crick paired ligation results are indicated in blue, and ligation products containing one or more mismatches are in orange.

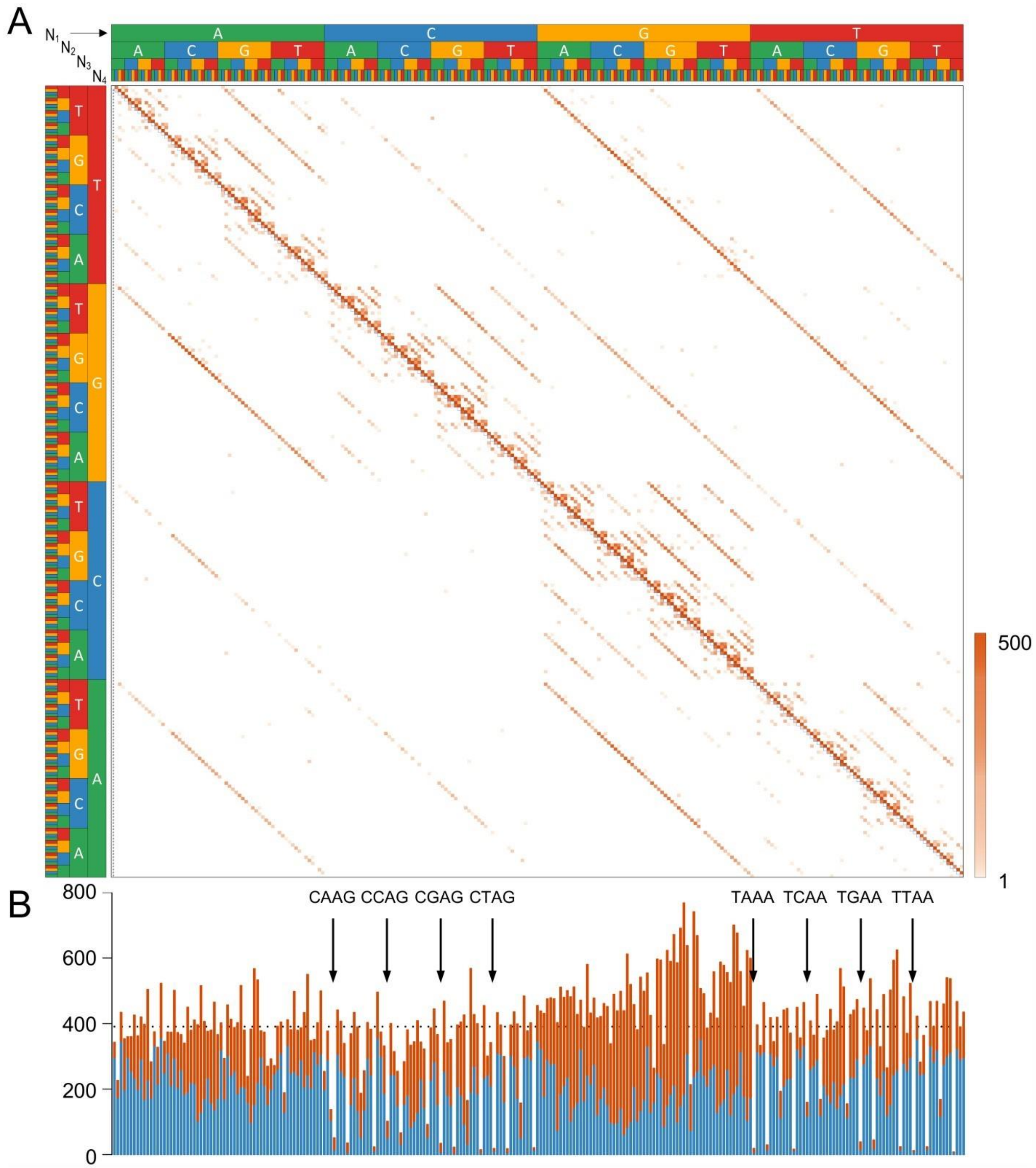

Figure S2H. Multiplex ligation assay results for the ligation of randomized four-base overhangs by hLig3 (PEG buffer). SMRT sequencing results for ligating 100 nM of the multiplexed four-base overhang substrate 1 h at 25°C, with 1.75 µM hLig3 in Quick Ligation Buffer. Observations have been normalized to 100,000 ligation events (File S1 for raw observation totals). (**A**) Frequency heat map of all ligation events (log-scaled). Overhangs are listed alphabetically left to right (AAAA, AAAC, AAAG …TTTG, TTTT) and bottom to top such that the Watson–Crick pairings are shown on the diagonal. (**B**) Stacked bar plot showing the frequency of ligation products containing each overhang, corresponding to each column in the heat map in (A). Fully Watson–Crick paired ligation results are indicated in blue, and ligation products containing one or more mismatches are in orange.

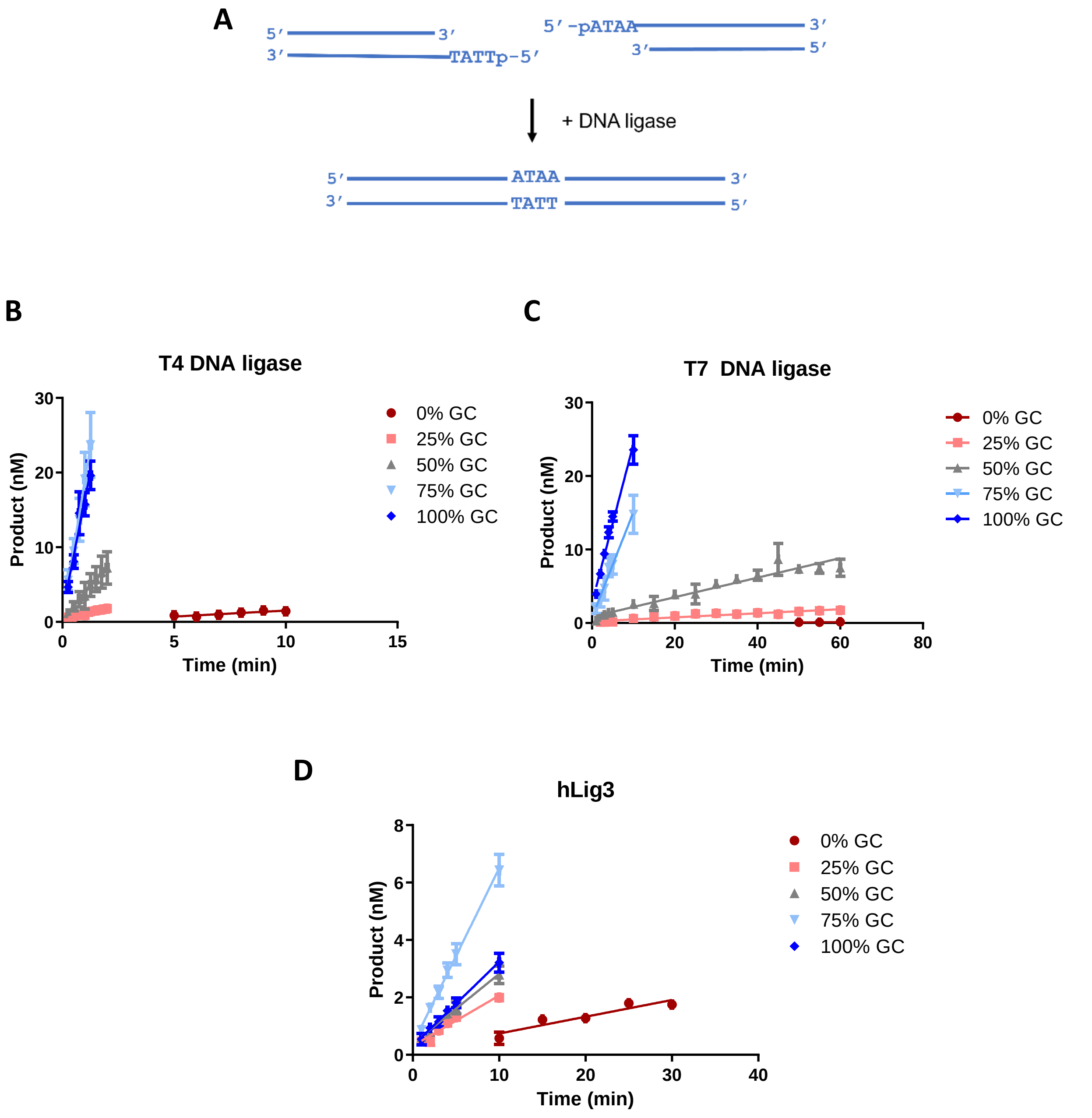

Figure S3. Ligation time courses with defined overhang substrates. A. Example schematic of the reaction of oligo substrate pairs (100 nM) with 5′ four-base overhangs of defined sequence with various DNA ligases (1 nM). Substrate sequences are detailed in Table S3 and substrates were FAM labeled on the 3' end of the phosphorylated strand. Initial velocity was determined by fitting the linear portion of the data with a linear regression up to a maximum of 25% product formation. Data points are the average of four replicates. B. Timepoints for T4 DNA ligase reaction were 15 s, 30 s, 45 s, 60 s, 75 s, 90 s, 105 s, 2 m, 3 m, 4 m, 5 m, 6 m, 7 m, 8 m, 9 m, 10 m. C. Timepoints for T7 DNA ligase reaction were 1 m, 2 m, 3 m, 4 m, 5 m, 10 m, 15 m, 20 m, 25 m, 30 m, 35 m, 40 m, 45 m, 50 m, 55 m, 60 m. D. Timepoints for hLig3 reaction were 1 m, 2 m, 3 m, 4 m, 5 m, 10 m, 15 m, 20 m, 25 m, 30 m, 35 m, 40 m, 45 m, 50 m, 55 m, 60 m.

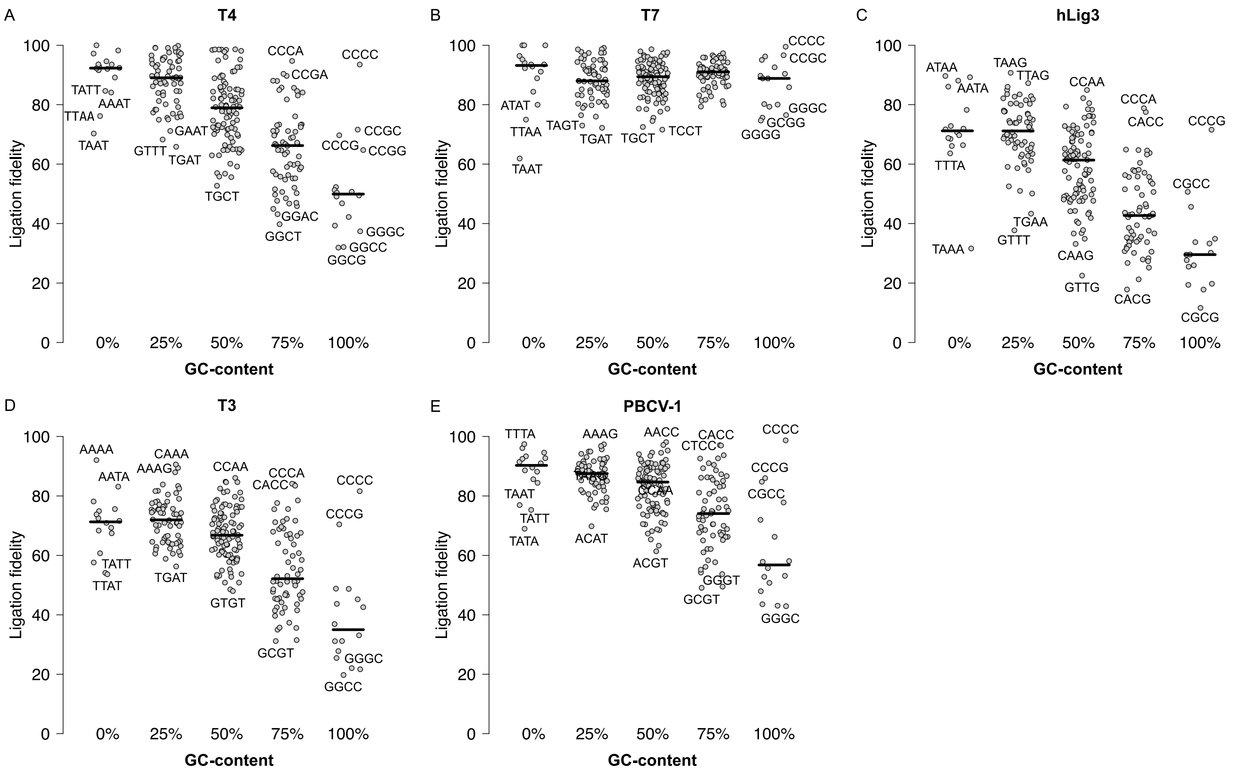

Figure S4. Ligation fidelity of individual overhangs according to GC content. For each DNA ligase tested, overhangs were grouped by GC content from 0% to 100% and their respective fidelities were plotted along Y-axis. Results of statistical significance testing are summarized in Table S2. This figure was generated from the same data as shown in Figure 3.

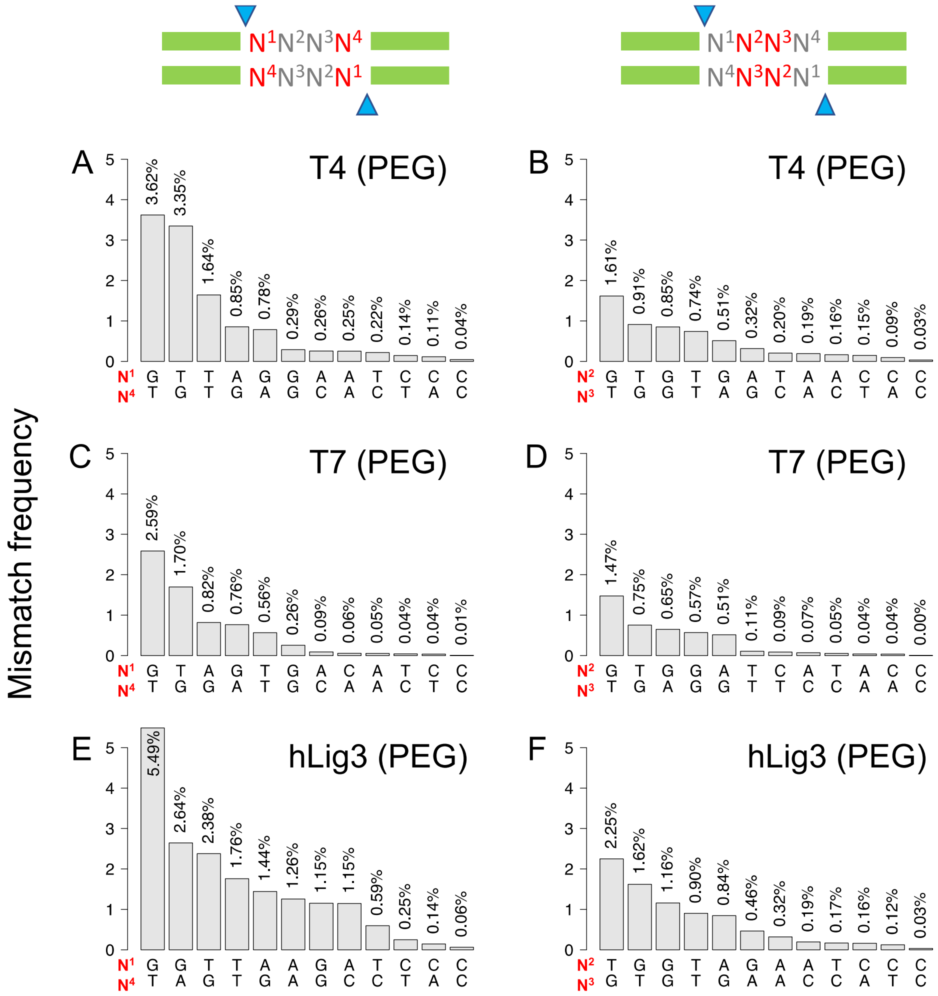

Figure S5. Positional mismatch profiles for ligations in PEG containing buffer. The frequency of each possible mismatched base pair mismatches by position was observed for ligation of four-base overhangs. The results shown are for SMRT sequencing of ligation reactions with 100 nM of the multiplexed four-base overhang substrate and 1.75 μM T4 DNA ligase, T7 DNA ligase, or hLig3 incubated 1 h at 25°C in Quick Ligation Buffer (Figures S2F-S2H and File S1 for raw data). (A, C, and E) show the results for the edge position (N1:N4); (B, D, and F) show the results for the middle position (N2:N3). The overhang positions (N^1, N^2, N^3, N^4) are numbered from 5′- to 3′- for each strand. Each position in N^1:N^4 and N^2:N^3 refers to bases in opposite strands. Note that as strand designation is arbitrary, all ligation products were counted in both orientations (top-to-bottom and bottom-to-top strand).

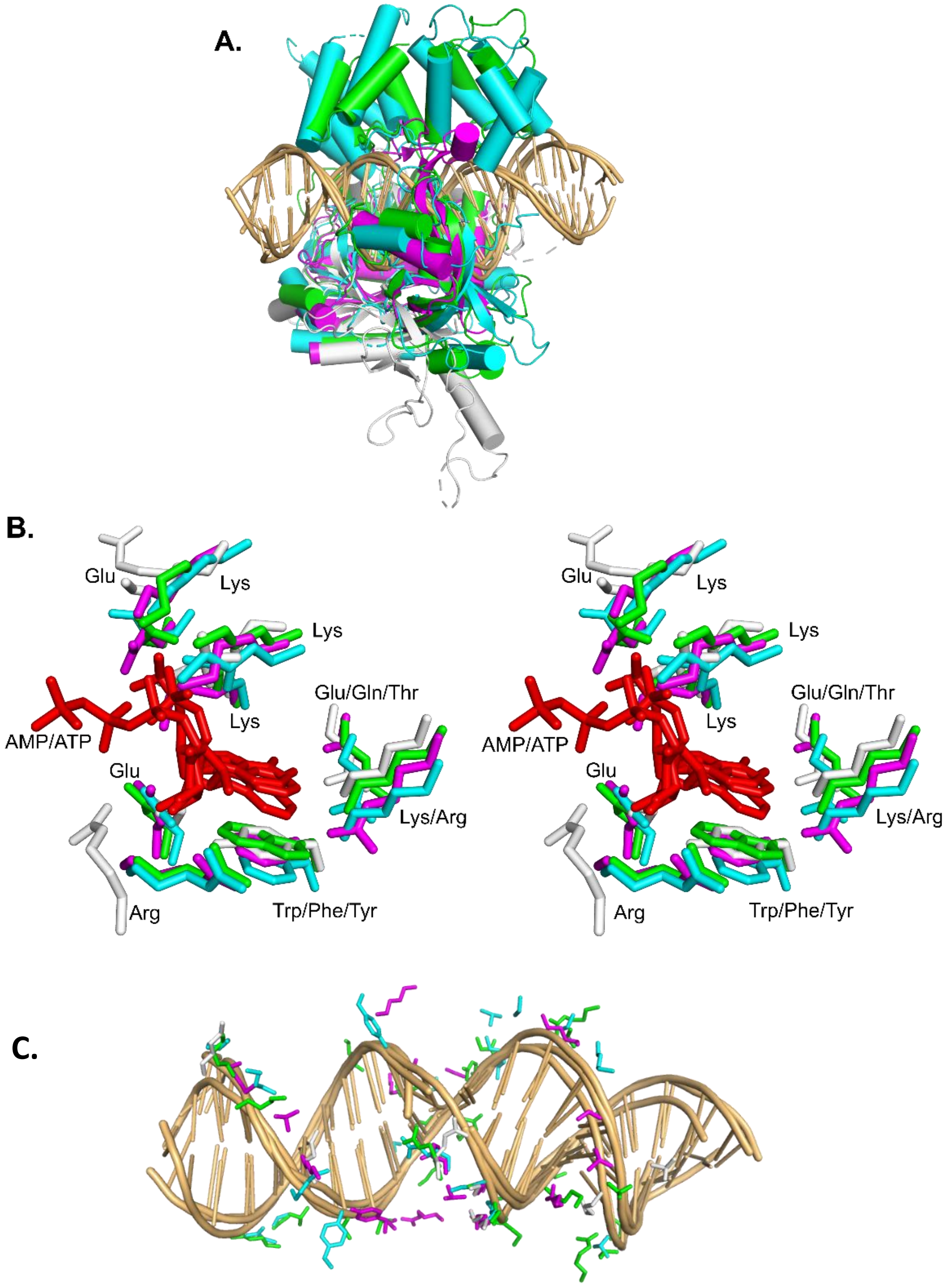

Figure S6. Comparison of DNA ligase structures. A. Overlay of T4 DNA ligase/DNA complex (PDB: 6DT1, green), hLig3/DNA complex (PDB: 3L2P, cyan), PBCV-1 DNA ligase/DNA complex (PDB: 2Q2U, magenta), and apo T7 DNA ligase (PDB: A10I, grey) with nicked DNA shown in gold where present. B. Overlayed active site residues of ligases. AMP/ATP is shown in red. C. DNA contacting residues from T4, PBCV, and hLig3. Interaction was defined as a distance of 3.6 Å or less for polar contacts. Note: there is no crystal structure available for T3 DNA ligase.
